# Demonstrating the reliability of in vivo metabolomics based chemical grouping: towards best practice

**DOI:** 10.1007/s00204-024-03680-y

**Published:** 2024-02-18

**Authors:** Mark R. Viant, E. Amstalden, T. Athersuch, M. Bouhifd, S. Camuzeaux, D. M. Crizer, P. Driemert, T. Ebbels, D. Ekman, B. Flick, V. Giri, M. Gómez-Romero, V. Haake, M. Herold, A. Kende, F. Lai, P. E. G. Leonards, P. P. Lim, G. R. Lloyd, J. Mosley, C. Namini, J. R. Rice, S. Romano, C. Sands, M. J. Smith, T. Sobanski, A. D. Southam, L. Swindale, B. van Ravenzwaay, T. Walk, R. J. M. Weber, F. M. Zickgraf, H. Kamp

**Affiliations:** 1 Phenome Centre Birmingham, University of Birmingham, Edgbaston, Birmingham, B15 2TT UK; 2 Amsterdam Institute for Life and Environment (A-LIFE), Vrije Universiteit Amsterdam, De Boelelaan 1085, 1081 HV Amsterdam, The Netherlands; 3 Division of Systems Medicine, Department of Metabolism, Digestion and Reproduction, Imperial College London, Hammersmith Hospital, Du Cane Road, London, W12 0NN UK; 4 European Chemicals Agency, Telakkakatu 6, FI-00121 Helsinki, Finland; 5 Department of Metabolism, Digestion and Reproduction, National Phenome Centre, Imperial College London, London, W12 0NN UK; 6 Division of Translational Toxicology, National Institute of Environmental Health Sciences, Research Triangle Park, NC 27709 USA; 7 BASF Metabolome Solutions GmbH, Tegeler Weg 33, 10589 Berlin, Germany; 8 Center for Environmental Measurement and Modeling, Environmental Protection Agency, Athens, GA 30605 USA; 9 BASF SE, Carl-Bosch-Str 38, 67056 Ludwigshafen, Germany; 10 Syngenta, Jealott’s Hill International Research Centre, Bracknell, RG42 6EY UK; 11 Present Address: NUVISAN ICB GmbH, Toxicology, 13353 Berlin, Germany; 12 Present Address: Environmental Sciences Consulting, 67122 Altrip, Germany

**Keywords:** Metabolomics, Reproducibility, Standardisation, Validation, OECD, Guidance

## Abstract

While grouping/read-across is widely used to fill data gaps, chemical registration dossiers are often rejected due to weak category justifications based on structural similarity only. Metabolomics provides a route to robust chemical categories via evidence of shared molecular effects across source and target substances. To gain international acceptance, this approach must demonstrate high reliability, and best-practice guidance is required. The MetAbolomics ring Trial for CHemical groupING (MATCHING), comprising six industrial, government and academic ring-trial partners, evaluated inter-laboratory reproducibility and worked towards best-practice. An independent team selected eight substances (WY-14643, 4-chloro-3-nitroaniline, 17α-methyl-testosterone, trenbolone, aniline, dichlorprop-p, 2-chloroaniline, fenofibrate); ring-trial partners were blinded to their identities and modes-of-action. Plasma samples were derived from 28-day rat tests (two doses per substance), aliquoted, and distributed to partners. Each partner applied their preferred liquid chromatography–mass spectrometry (LC–MS) metabolomics workflows to acquire, process, quality assess, statistically analyze and report their grouping results to the European Chemicals Agency, to ensure the blinding conditions of the ring trial. Five of six partners, whose metabolomics datasets passed quality control, correctly identified the grouping of eight test substances into three categories, for both male and female rats. Strikingly, this was achieved even though a range of metabolomics approaches were used. Through assessing intrastudy quality-control samples, the sixth partner observed high technical variation and was unable to group the substances. By comparing workflows, we conclude that some heterogeneity in metabolomics methods is *not* detrimental to consistent grouping, and that assessing data quality prior to grouping is essential. We recommend development of international guidance for quality-control acceptance criteria. This study demonstrates the reliability of metabolomics for chemical grouping and works towards best-practice.

**Supplementary Information:**

The online version contains supplementary material available at 10.1007/s00204-024-03680-y.

## Introduction

Metabolomics has reached a critical point in determining its value to regulatory toxicology. Building on 20 years of research, metabolomics data are now starting to be used in industry to support grouping and read-across (van Ravenzwaay et al. 2016; Sperber et al. 2019), several other applications of metabolomics in regulatory toxicology have been described (Viant et al. 2019), and the OECD Omics Reporting Framework (OORF) has been developed (Harrill et al. 2021). Further ongoing initiatives are focused on developing reporting for ‘omics-based grouping, constructing a framework for ‘omics data interpretation, and the design of smart in vivo studies incorporating ‘omics technologies. Under Europe's chemicals legislation REACH, grouping and read-across (OECD 2017) is the most widely used ‘alternative method’ in chemical risk assessment for filling data gaps in chemical safety dossiers with existing in vivo toxicity data. A Read-Across Assessment Framework (RAAF) has been published by the European Chemicals Agency (ECHA) to provide a consistent and structured approach to the scientific evaluation of read-across justifications (ECHA 2017a). However, many dossiers are rejected by the regulator due to quality deficiencies, including poor documentation, lack of or low quality of supporting data, and shortcomings in the toxicological hypothesis (ECHA 2017b). This has prompted an effort to increase the confidence in grouping and read-across by integrating evidence of similar biological responses to chemical exposure. The first time that metabolomics was proposed to address this challenge was more than a decade ago (van Ravenzwaay et al. 2012). Since then, although recognition of the value of metabolomics as a New Approach Methodology (NAM) for chemical grouping to support read-across has increased (Sperber et al. 2019; Viant et al. 2024) there remains a particular need to assess its interlaboratory reproducibility when used for this specific purpose, as a key step towards its validation.

The overarching aim of this blinded ring trial was to demonstrate the reliability (specifically laboratory reproducibility) of liquid chromatography–mass spectrometry (LC–MS) metabolomics when applied to chemical grouping using rat plasma, and to work towards deriving best practice for the use of metabolomics in this regulatory application. The ring trial, named MATCHING (MetAbolomics ring Trial for CHemical groupING), comprised an international consortium of six industrial, government and academic metabolomics ring-trial partners, BASF SE who led on the in vivo exposure study, and ECHA as an independent advisor. Specifically, the roles of ECHA were to contribute to the chemical selection (together with BASF SE), ensure the blinding conditions of the study were met, and to that end all partners sent their results to ECHA before the results were disclosed to the other partners. The roles of the eight organisations are summarized in Table S1 (Online Resource 1). To ensure the greatest relevance to the chemical industries and regulatory authorities, the ring trial was embedded within an in vivo study conducted in accordance with OECD Test Guideline 407 (OECD 2008) with minor modifications (e.g., only two dose levels per compound). While several metabolomics ring trials have been reported previously (e.g., Thompson et al. 2019; Lin et al. 2020), they focus on the nuances of analytical reproducibility (e.g., the measurement precision of metabolites). Unique to this study is that we assess metabolomics reproducibility across multiple laboratories in terms of the consistency of the downstream findings and conclusion of regulatory relevance. Here, by ‘conclusion of regulatory relevance’, we mean the conclusions drawn on the membership of chemicals within groups as derived from the similarities of the metabolic responses to those multiple chemicals. This is the first ring trial based on chemical grouping in a regulatory context. Ultimately, a demonstration of high reproducibility of the conclusion of regulatory relevance would contribute towards the validation of this ‘omics approach for grouping (OECD 2005) and thereby its wider uptake by the chemical industry for this regulatory application.

The first objective was to design the blinded in vivo exposure study, including defining the number of chemical groups and identities of eight test substances, and then conduct a 28-day rat study to prepare a consistent set of plasma samples to be aliquoted and distributed to the metabolomics ring-trial partners. The test substances were selected by BASF SE and ECHA (guided by the MetaMap^®^Tox database), and all ring-trial partners and the laboratory team conducting the exposure study at BASF SE were fully blinded to their identities, modes of action (MoA), and the number of chemical groups (i.e., MoA categories). BASF SE also checked that a metabolomics analysis of the study samples yielded the anticipated grouping of test substances, thereby defining the ‘target result’ for the six blinded ring-trial partners. Ultimately, this design would allow the MATCHING team to draw conclusions on the ring-trial accuracy (relative to the MoA classifications within BASF SE’s MetaMap^®^Tox database of metabolomics signatures (van Ravenzwaay et al. 2015)) as well as reproducibility. The second objective was for each of the blinded ring-trial partners to acquire, process and analyze their metabolomics data, with appropriate quality-control (QC) samples, and then attempt to group the substances based on the similarities of the endogenous metabolic responses. While all partners utilized LC–MS metabolomics, i.e., the most widely used analytical platform in metabolomics as evidenced by international surveys (Weber et al. 2017), they were able to select their preferred protocols for the analytical and statistical procedures. By including method heterogeneity, this design would help to ensure that the findings from the study would be applicable to real-world applications of this ‘omics technology. Each ring-trial participant was instructed to report their chemical grouping results and conclusions to ECHA to ensure the blinding conditions of the study were met, including testing the OECD Omics Reporting Framework. Next, the results were revealed to the partners to assess whether they came to the same chemical groupings. The final objective was to propose best practices for executing bioactivity-based grouping using metabolomics data, considering both the processes and QA/QC criteria. A blinded attempt to derive consistent biomarker signatures associated with each chemical group was beyond the scope of this first study. Ultimately, the ring trial sought to determine whether this technology is fit-for-regulatory-purpose (by demonstrating a high consistency of chemical grouping), or whether refinements in analytical or data analysis practices are needed.

## Materials and methods

### Ring-trial design

The study comprised three objectives, which were mapped directly to work package activities (Figure S1, Online Resource 1). Work package 1 included the selection of eight test substances by BASF SE and ECHA (described in Sect. "Test substance selection"), the 28-day rat exposures and plasma sampling by BASF SE (Sect. "Animal exposures and plasma sampling"), and the initial evaluation of those samples by BASF SE to ensure that the similarities of the metabolic responses resulted in the anticipated chemical grouping (Sect. "Evaluation of quality of plasma samples to ensure anticipated chemical grouping"). During work package 2, the six ring-trial partners worked independently to prepare samples, acquire LC–MS metabolomics data, process and statistically analyze the data, and then report their chemical grouping results to ECHA. Throughout, the ring trial, partners were blinded to the substance identities, their mode(s) of action, the number of chemical groups (i.e., MoA categories), and whether the MoAs and number of chemical groups were consistent between male versus female rats. Upon receiving the plasma samples, the ring-trial partners were made aware of the 180 sample identifiers (Table S2, Online Resource 1) that were named according to a defined convention (Table S3, Online Resource 1). Hence the partners were not blinded to the sex of the animal samples, nor to the anonymized treatment group (test substance number) and dosing level. Only after a ring-trial partner formally reported their findings to ECHA were they unblinded to the results that ECHA had received from other partners. Work package 3 then focused on the collective analysis of the results.

### Test substance selection

The test substances for the ring trial were selected by a small team at BASF SE and ECHA. Based on plasma metabolomics data available for more than 750 compounds in BASF’s database MetaMap^®^Tox (van Ravenzwaay et al. 2015), a set of 29 substances were selected that are well described regarding their toxicity and show effects on the metabolome of differing magnitudes, for eight different MoAs. From this group, a short list of ten substances was selected, covering three MoAs that are relevant for chemical safety assessment. Additionally, substances with different potencies were included, which in principle could be grouped according to their LC–MS metabolomics data, with the low potency substances acting as a more stringent test of the ability of the ring-trial partners to group successfully. From these ten substances, eight were selected based on their commercial availability and ease of handling (Table 1).

**Table 1:** Test substances with their route of administration, dosing vehicle, dose levels and known MoA, and hence MoA-defined grouping

Code	Test substance	CAS no.	MoA	High dose	Low dose	Vehicle
TS1	WY-14643	50892-23-4	PP	1200 ppm	400 ppm	In diet
TS2	4-Chloro-3-nitroaniline	635-22-3	Anaemia	90 mg/kg b.w	30 mg/kg b.w	In corn oil
TS3	17α-Methyl-testosterone	58-18-4	AR	80 mg/kg b.w	20 mg/kg b.w	In corn oil
TS4	Trenbolone	10161-33-8	AR	30 mg/kg b.w	10 mg/kg b.w	In corn oil
TS5	Aniline	62-53-3	Anaemia	100 mg/kg b.w	10 mg/kg b.w	In aqua bidest
TS7	Dichlorprop-p	15165-67-0	PP	2250 ppm	1000 ppm	In diet
TS8	2-Chloroaniline	95-51-2	Anaemia	160 mg/kg b.w	40 mg/kg b.w	In corn oil
TS9	Fenofibrate	49562-28-9	PP	400 mg/kg b.w	100 mg/kg b.w	Drinking water containing 0.5% CMC

Due to limited commercial availability, substance TS6 was replaced by the backup substance TS9

*PP* peroxisome proliferation, *AR* androgen receptor activity

### Animal exposures and plasma sampling

The animal study was conducted by BASF SE according to the German Animal Welfare legislation in an AAALAC (Association for Assessment and Accreditation of Laboratory Animal Care) certified laboratory, described in Section S1 (Online Resource 1). In brief, Wistar rats (Crl:WI(Han)) were obtained from Charles River Laboratories, Sulzfeld, Germany. The animals were housed together (5 animals per cage) in polysulfonate cages, with dust-free wooden bedding, and wooden gnawing blocks for environmental enrichment. The animals were kept under fully standardized conditions and diet and drinking water were available ad libitum (except before blood sampling). Groups of five male and female rats were treated with the eight ring-trial chemicals at each of two dose levels for 28 days. Ten animals per sex served as a control group. Dose levels were selected based on previous 28-day repeated dose studies, with the high dose chosen to induce clear effects without causing suffering to the animals and not exceeding the maximum tolerated dose for a 28-day study (Table 1). Parts of the study, i.e., clinical examinations, clinical chemistry and sampling for histopathology, were conducted in accordance with the OECD Test Guideline No. 407: Repeated Dose 28-day Oral Toxicity Study in Rodents (OECD 2008). The following parameters were determined: mortality, clinical signs of toxicity, body weight, food consumption, haematology, organ weights, and macroscopic pathology. Tissues for potential histopathological examinations were fixed and stored. On study day 21, blood was taken from non-fasted animals for measuring the haematological parameters. Individual blood samples for metabolomics were taken from fasted animals by puncturing the retrobulbar venous plexus on study days 7 and 14 for all test groups under isoflurane anaesthesia, and on study day 28 after decapitation under isoflurane anaesthesia. Plasma samples generated from the blood taken on day 28 from each animal were used for this ring trial (see Section S1 (Online Resource 1) for details). All plasma samples were stored in Eppendorf tubes, covered with an N_2_ atmosphere, at − 80 °C. The plasma samples were sent to BASF Metabolome Solutions (BMS) on dry ice, who subsequently distributed them to all partners on dry ice with temperature monitors.

### Evaluation of quality of plasma samples to ensure anticipated chemical grouping

Before the samples were sent to the ring trial partners, their quality was evaluated by (a) BMS’ quality control procedures, and (b) BASF through comparison with BASF’s in-house database MetaMap^®^Tox. Quality control involved the analysis of the variation and completeness of technical controls, completeness at metabolite and group level, linearity of the response per metabolite based on a dilution series, as well as uni- and multivariate checks for outliers and within group consistency. The comparison with the MetaMap^®^Tox database was to ensure that the chemical groups and potential MoAs and substance classes of the eight test substances could be identified as expected. This evaluation was conducted blindly, comprising three steps. First, substances were grouped using ‘treatment correlation’. Next, based on the identified groups, common patterns were analysed by applying ‘pattern ranking’, allowing identification of potential MoAs. Finally, to uncover the potential substance classes, the treatment correlation and pattern ranking results were combined.

Treatment correlation compares the metabolome of a test substance with the metabolomes of all other substances in the MetaMap^®^Tox database, thereby identifying substances that show a similar metabolome profile to other substances. Threshold values of 0.40 for male rats, and 0.50 for females, represented approximately the 95th percentile of Pearson correlation coefficients between all pairs of treatments in the database, hence correlation coefficients above these values were considered as showing high similarity between two treatments (van Ravenzwaay et al. 2015). To group the eight substances, a treatment correlation analysis was performed per substance, separately for low and high dose as well as for males and females, using the thresholds above. The top-ranking compounds were considered as a group, especially if a consistent grouping could be derived based on the independent analyses of low and high doses, as well as males and females.

In addition to treatment correlation, which uses *t*-values from all metabolites for pairwise comparison of individual treatments, pattern ranking was applied as another standard evaluation method. The latter approach assumes that substances that induce a specific form of toxicity share a common set of metabolite changes, referred to as a “pattern”. Contrary to the treatment correlation, pattern ranking is (a) only using the subset of metabolites that is consistently and significantly changed across the substances representing the pattern, and (b) evaluating the test substance against a set of pattern substances where the median uncentered correlation is determined. Comparing the metabolic response to a test substance with a list of patterns of metabolite changes predictive of a particular MoA is defined as “pattern ranking”, and can result in matches, weak matches, equivocals or mismatches, based on the overlap of significantly changed metabolites in the right direction (Kamp et al. 2012a, b; van Ravenzwaay et al. 2015, 2016; Sperber et al. 2019). Pattern ranking was applied to identify the MoA of each test substance, and only patterns with matches or weak matches resulted in a predicted MoA.

By combining the results from treatment correlation and pattern ranking, the potential substance classes tested in the ring trial were predicted. The results from this blinded analysis were then shared with an unblinded scientist from BASF SE (Kamp), who checked whether the test substances grouped as anticipated.

### Acquisition, processing and quality assessment of LC–MS metabolomics data

Each ring-trial partner independently extracted polar and lipophilic metabolites from the plasma samples and then acquired and processed hydrophilic interaction liquid chromatography (HILIC) and reverse-phase (‘lipid’) LC–MS metabolomics datasets, respectively, all according to their own standard operating protocols. This approach introduced a realistic degree of heterogeneity into the ring trial as would be encountered in the regulatory use of metabolomics data for bioactivity-based grouping, summarised for the six partners in Table S4 (Online Resource 1), including the use of targeted, untargeted, and hybrid (combining targeted and untargeted) methods which have been described previously (Lewis et al. 2016; Mosley et al. 2018; Sands et al. 2019; Southam et al. 2020; Lloyd et al. 2021; Fu et al. 2022; Sostare et al. 2022; Viant et al. 2023; Kende et al. 2023; Wang et al. 2023). Detailed descriptions (and further references) of the methods for sample extraction, acquisition, and processing of the LC–MS metabolomics data, by each partner, are provided in Section S2. Acquisition and processing of LC–MS metabolomics data (Online Resource 1). The main exception to this unconstrained approach was the mandatory inclusion of intrastudy QC samples by all partners, as described in the MEtabolomics standaRds Initiative in Toxicology (MERIT) best practice guidelines (Viant et al. 2019). Additionally, it was mandatory for each partner to create and measure a process (extraction) blank. While partners were allowed to select their own data processing workflows, two steps were mandatory. First, the removal of features present in process (extraction) blanks, and secondly the removal of features not present in control samples to enable the analysis to focus on endogenous metabolites and lipids only. Each partner quality-assessed their data (Section S3. Intrastudy QC results, Online Resource 1) to determine whether they should progress to the chemical grouping.

### Statistical analysis of metabolomics data to group chemicals

Ring-trial partners independently determined their preferred univariate and/or multivariate statistical approaches for grouping the eight substances based on the similarities of the metabolic responses. As for the analytical methods, this unconstrained approach was used to ensure the conclusions from the study would be applicable to real-world applications. Descriptions of the univariate and multivariate statistical methods used by the partners for grouping the substances and estimating uncertainty in their grouping are provided in Section S4. Analysis of metabolomics data to group chemicals (Online Resource 1), including hierarchical cluster analysis, correlation analysis, multivariate visualisation, (orthogonal) partial least squares discriminant analysis, linear discriminant analysis and bootstrapping approaches. In contrast, the format for reporting the study findings to the independent advisor (ECHA) was constrained, to ensure that the results could be readily compared across the six partners. Specifically, each partner was requested to summarize their analytical and computational workflows, analytical data quality, and grouping results for male and female rats, separately, in a Microsoft PowerPoint presentation.

### Statement on data availability

The raw experimental Metabolomics data supporting the findings of the ring trial are available in the MetaboLights repository (https://www.ebi.ac.uk/metabolights/) under the identifier MTBLS8274. In accordance with the FAIR (Findable, Accessible, Interoperable, and Reusable) principles (Wilkinson et al. 2016; Jacobsen et al. 2020), the metadata associated with the datasets, including sample information, experimental details, and analytical and computational methods, is provided. By making the raw data accessible, we aim to promote collaboration and facilitate future research efforts.

## Results and discussion

### Chemical selection and confirmation of categories

**Fig. 1:**
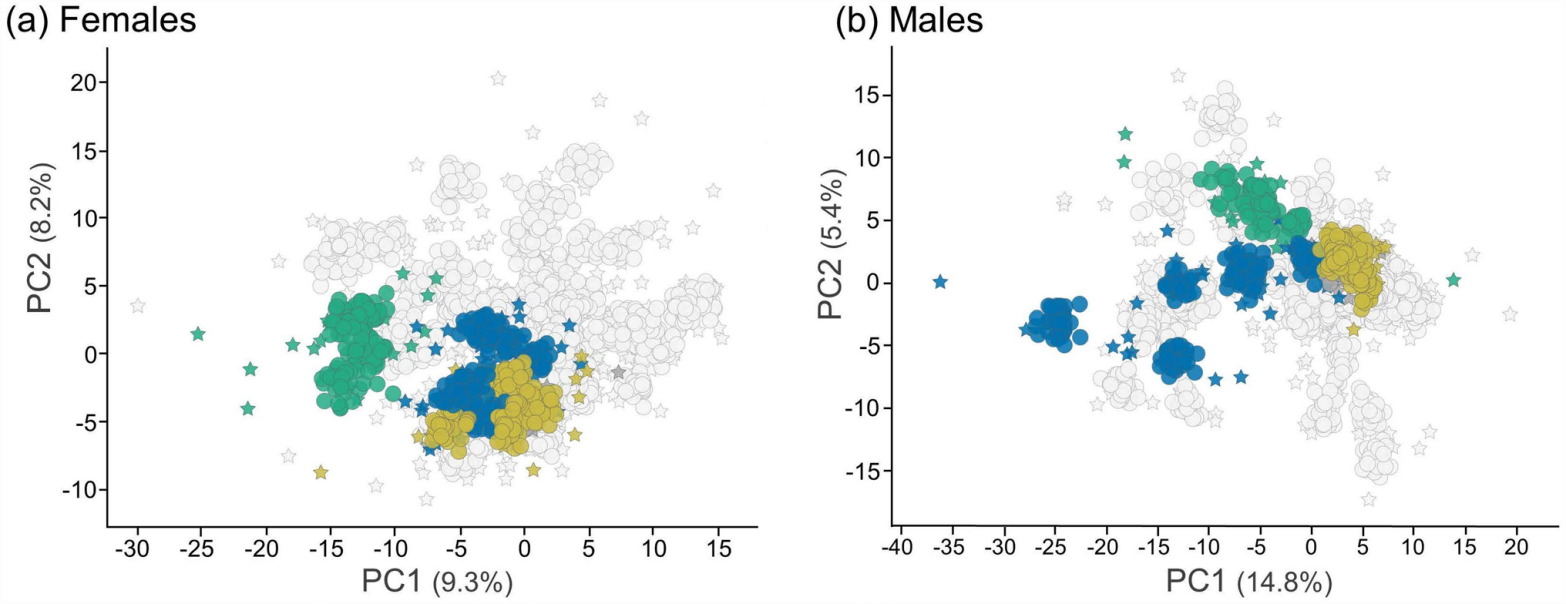
Mapping of the 8 test substances to the MetaMap^®^Tox database demonstrates that the three MoA categories are only moderately separated in metabolomics (biological) response space. Results show bootstrap PCA scores plots for **a** female, **b** male rats using historic MetaMap^®^Tox metabolomics data for 29 substances (including the 8 ring-trial test substances), each at two doses, and spanning 8 known MoAs (plus no-treatment as a negative control). Input metabolomics data were bootstrapped to generate 100 pseudo-samples per treatment (for historic and new study data) that were split into 70 training and 30 test samples; the PCA model was calculated on the training set (historic data only, not shown) and then used to predict the 30 test samples per treatment (for historic and new study data). Historical data are shown in grey (for all 29 substances) to denote the biological response space, and the 8 test substances in the ring trial are coloured to represent the three MoA categories (gold: anaemia, green: androgen receptor activity, blue: peroxisome proliferator). Input metabolomics data are represented by stars, bootstrapped test data by circles

The eight chemicals that were selected by BASF SE and ECHA (according to the criteria defined in Sect. "Test substance selection"), and to which all ring-trial partners were blinded, are listed in Table 1 together with their MoAs. Given that the extent to which these MoAs differed would have a significant impact on the ease, or difficulty, of grouping the chemicals. Figure 1 shows the eight substances within the context of the MetaMap^®^Tox chemical space that was evaluated during test substance selection (Sect. "Test substance selection"), thereby confirming that neither extremely different, nor extremely similar, MoAs were selected. Following (blinded) metabolomics data acquisition by BMS, one of the (blinded) BASF SE team utilised MetaMap^®^Tox software to analyse the metabolomics data (Fig. S2, Online Resource 1), providing the results to another member (unblinded) of the BASF SE team who could confirm that the rat plasma samples yielded the anticipated grouping of test substances (as defined in Table 1). This grouping defined the ‘target result’ for the six blinded ring-trial partners, allowing this study to determine both the accuracy and reproducibility of bioactivity-based grouping using metabolomics data.

### Acquisition, processing, and quality assessment of metabolomics data

**Fig. 2:**
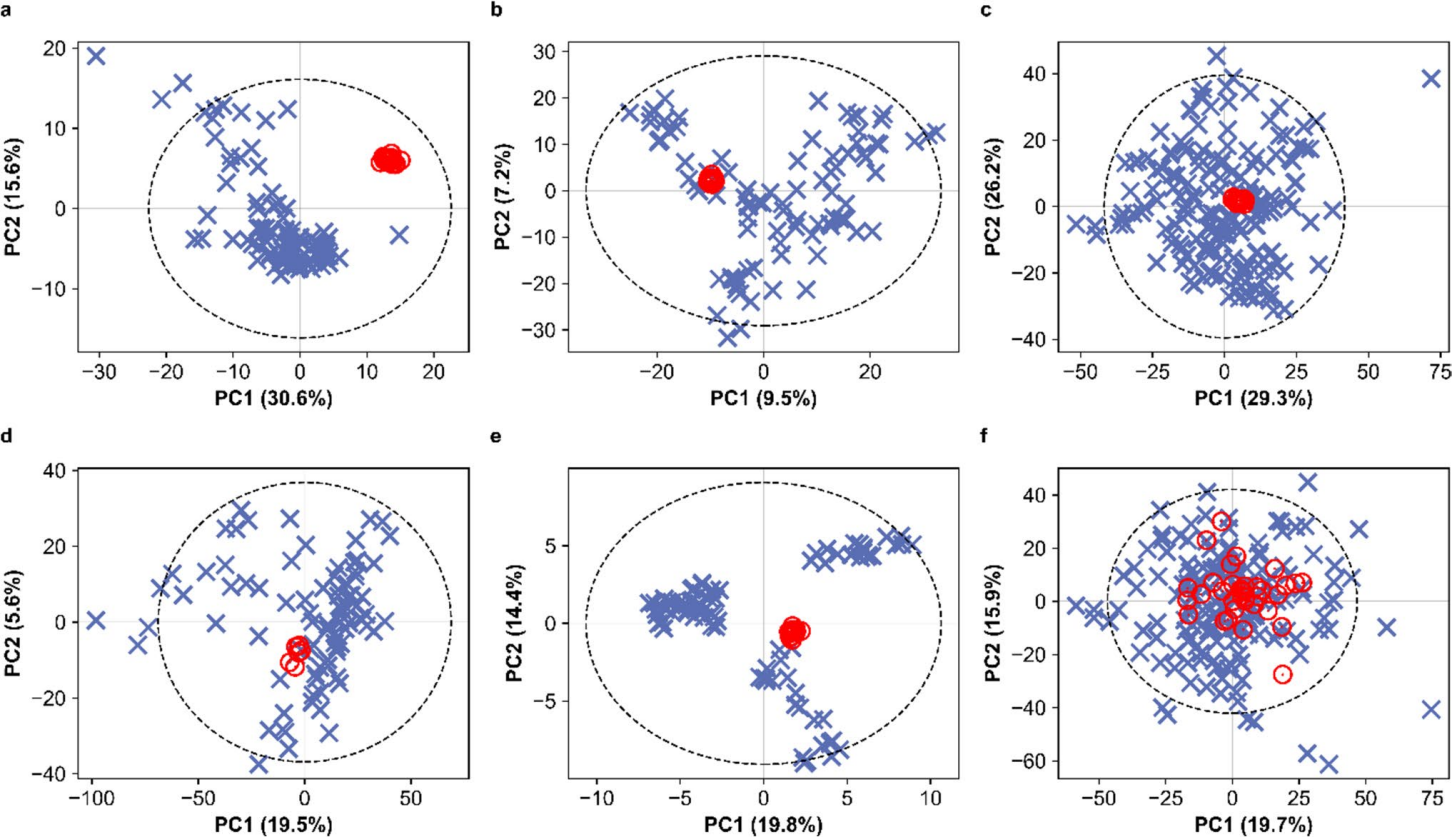
Composite of exemplar PCA scores plots (with Hotelling’s T2 ellipse at 95% confidence interval) from each blinded partner for assessment of LC–MS metabolomics data quality after all processing steps, prior to statistical analysis. Plots demonstrate the low technical variability of the intrastudy QC samples (red circles) relative to the larger biological variability of the study samples (treated and control animals, blue crosses) across all partners. The much higher technical variability relative to biological variability observed by partner RP8 (panel **f**), compared to other partners, is clearly evident. Included data are partner-protocol specific: **a** RP1, all LC–MS assays, male animals; **b** RP4, one HILIC LC–MS assay, female animals; **c** RP5, one lipid LC–MS assay, both sexes; **d** RP6, all LC–MS assays, male animals; **e** RP7, one HILIC LC–MS assay, female animals; **f** RP8, two lipid LC–MS assays, both sexes

As described in Methods, each ring-trial partner was allowed to select their preferred methods to extract the plasma samples and then acquire and process the metabolomics data. This approach introduced a realistic degree of heterogeneity into the ring trial to ensure that the study findings could be broadly applied. However, it was mandatory for all partners to assess the quality of their processed data using intrastudy QC samples and determine whether it was of sufficient quality to proceed to the grouping. Representative intrastudy QC results for all six partners are summarized graphically in Fig. 2 and presented in Sect. "Results and discussion". Intrastudy QC results and Table S5 (Online Resource 1). These results illustrate how the technical variation in feature intensities, arising from the repeated measurement of a series of equivalent intrastudy QC samples, is typically low compared to the variation in feature intensities across the biological study samples. However, this was not the case for one of the six partners, who reported a large median RSD of the intrastudy QC measurements (Parsons et al. 2009), indicating high technical variation. Having completed the data processing and quality assessment, only five of the six ring-trial partners concluded that they had achieved sufficiently high analytical quality, based on their own criteria from historical experience, to proceed to the bioactivity-based grouping. While investigating the origin(s) of the high analytical variability observed by the sixth partner is beyond the scope of this publication, these results highlight the importance of calculating and reporting intrastudy QC metrics in regulatory toxicology to check that metabolomics datasets are of sufficiently high quality to ensure reliable findings (Viant et al. 2019). Reporting intrastudy QC results is required by the OECD Omics Reporting Framework (Harrill et al. 2021).

### Bioactivity-based grouping by each ring-trial partner

**Fig. 3:**
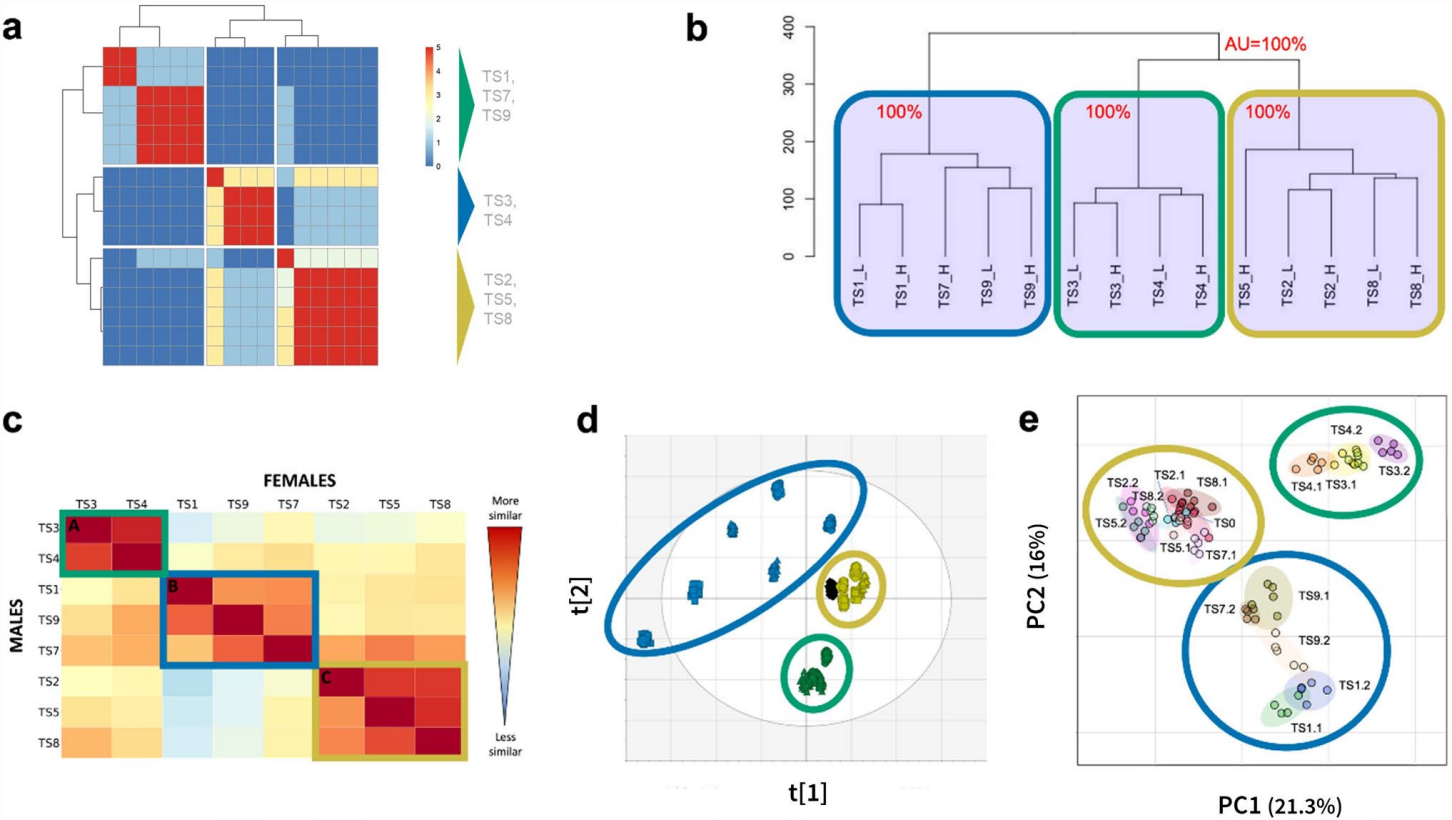
Composite of graphical visualisations of the chemical grouping results submitted to the European Chemicals Agency, one from each of the five blinded partners in the metabolomics ring trial whose dataset passed quality control. Results for each partner show consistent grouping of test substances, comprising Group A: TS1, TS7 and TS9 (highlighted in blue); Group B: TS2, TS5 and TS8 (gold); Group C: TS3 and TS4 (green). Approaches to group test substances were partner-protocol specific: **a** RP1, pairwise correlation of t-value profiles (treatment vs. control), all LC–MS assays, for male animals; **b** RP4, hierarchical cluster analysis applied to t-values (treatment vs. control), *p* values calculated using a bootstrap procedure, all LC–MS assays, females only; **c** RP5, profile similarity matrix for females (top right) and males (bottom left), with red indicating a high profile similarity (i.e., high percentage of shared features between treatment groups) and yellow/pale blue indicating a low similarity, all LC–MS assays; **d** RP6, bootstrapped PCA scores plot with colours indicative of groups and controls in black, all LC–MS assays, males only; **e** RP7, PCA scores plot with colours indicative of groups, HILIC LC–MS assays, females only. For details, see Online Resource 1, Section S4. Analysis of metabolomics data to group chemicals

Five of the six blinded partners proceeded to group the test substances based on the similarities of the metabolomics responses, and then each submitted their results to ECHA. The approach used by each partner along with their findings, typically achieved by applying multiple statistical methods to gain higher confidence in the grouping, are described in Sect. "Conclusions". Analysis of metabolomics data to group chemicals (Online Resource 1). Graphical visualisations of the grouping results, one from each of the five blinded partners, are presented in Fig. 3. While this figure highlights the diversity of statistical methods employed, this did not impact on the ability of the five partners to achieve the same grouping results, which are summarized in Table 2. All five partners, whose datasets passed quality-control, correctly identified test substances 1, 7 and 9 in one group, substances 2, 5 and 8 in a second group, and substances 3 and 4 in a third group, for both male and female rats. Notably, a wide variety of metabolomics approaches (targeted, untargeted and hybrid data acquisition) using different analytical platforms (Orbitrap, QToF, QTrap), and LC columns and mobile phases, were used. The heterogeneity in LC–MS metabolomics methods resulted in datasets with varying number of metabolite features, ranging from several hundred to many thousands. Strikingly, all these approaches led to the same (and correct) grouping results, providing evidence of the effectiveness of metabolomics data for chemical grouping.

**Table 2:**
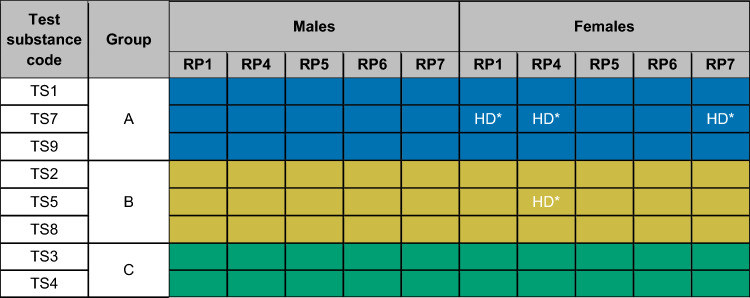
Summary of the chemical grouping results from the five blinded partners in the metabolomics ring trial whose datasets passed quality control, for both male and female rats. HD* indicates that the decision was based on the higher dose only

The substances selected for the ring trial by BASF SE and ECHA were deliberately chosen to exemplify some of the known technical challenges in chemical grouping/read-across, namely grouping across a potency range and grouping low toxicity chemicals. While the grouping of those test substances causing a strong effect on the metabolomic plasma profiles was conducted with confidence by the ring-trial partners, test substances inducing weaker effects were harder to assign to a group. The difference in potencies was clearly observable in the group comprising test substances 1, 7 and 9, where responses ranged from those similar to controls to very strong effects. In such cases, it can be challenging to differentiate potency and toxicological MoA when relying purely on statistical analysis. In contrast, test substances 3 and 4 induced strong metabolic effects and had very similar potencies. The group containing test substances 2, 5 and 8 generally caused a weaker effect on the plasma profiles, based on their similarity to the control group samples. Of particular note was the low dose group of substance 5, which caused such a subtle effect that some of the ring-trial partners excluded it from their statistical analysis, classifying it as a ‘non-responder’. This raises an important question in the derivation of best practices for grouping using ‘omics data, how to define and set the threshold for ‘responder’ vs. ‘non-responder’, i.e., the no observable metabolomic effect level (NOMEL), in a methodology agnostic way.

### Towards deriving best practice for bioactivity-based grouping using metabolomics data

Given that all five partners whose datasets passed quality-control correctly identified that the eight test substances group into three chemical categories, and correctly identified which substances were within each category, and correctly discovered this result for both male and female rats, we then reviewed all of the analytical and computational methods used by the partners to attempt to identify: (a) which methods were consistently used by the five partners that grouped the substances correctly, associating these methods with emerging best practice; (b) which methods were *not* consistently employed across these five partners, inferring that these particular methods do not need to be highly consistent to group substances accurately; and (c) whether QC assessments adequately differentiated the five partners who grouped consistently from the sixth laboratory that did not. While this study works towards describing best practices for bioactivity-based grouping using metabolomics data, this assessment of which methods were used does not imply that all other practices are necessarily unacceptable.

Assimilation of the six sets of methods was based around the modules within the new OECD Omics Reporting Framework (Harrill et al. 2021), utilising the ‘Data Acquisition and Processing Reporting Module for Mass Spectrometry’ (DAPRM-MS), the ‘Data Analysis Reporting Module for Multivariate Analysis (DARM-MVA), and the draft ‘Chemical Grouping-Application Reporting Module’ (CG-ARM) that is currently under development by the OECD Working Party for Hazard Assessment. Table S6 (Online Resource 2) provides a detailed assessment of the consistency of the analytical and computational methods used, while a summary of these findings is presented in Table 3. According to a series of *high-level method descriptions* (Table S6, Online Resource 2), every partner conducted ‘Sample processing’, ‘Data acquisition’, ‘Data preparation’, ‘Data cleaning’, ‘Data preprocessing’, and ‘Data quality assessment’, with the five partners who consistently grouped the substances also all applying ‘Metabolite feature annotation’, ‘Bioactivity-based grouping’ and ‘Report grouping results’; these latter three processes were not applicable to the sixth partner who stopped their analyses once it was determined that their QC criteria had not been passed.

**Table 3:** Summary of emerging best practices for (i) metabolomics data acquisition and processing, (ii) QA/QC practices to ensure high quality data, and (iii) bioactivity-based chemical grouping, structured according to the OECD Omics Reporting Framework (Harrill et al. 2021)

OORF reporting element	Used by no. of partners	Range of methods reported
**OORF Data Acquisition and Processing Reporting Module: acquisition**		
Extraction method	6	Polar—methanol (MeOH)/acetonitrile (ACN) OR ACN, Lipid—isopropanol (IPA), Combined—MeOH/dichloromethane/water/toluene
Extract concentration and reconstitution	6	Samples did not require drying and reconstitution, Lipid—sample concentration and solvent exchange
QC samples	6	Intrastudy QC (isQC) from pooled sample aliquots (m & f together), isQC from pooled sample extracts (m & f together), isQC from pooled sample extracts (m, f separately), Intralaboratory pooled QC (m & f together)
Process blank sample	6	Process blank filtering (dictated by ring-trial protocols)
Mass spectrometry assay type	6	Targeted with relative quantification (rel. quant.), Untargeted with rel. quant., Hybrid with rel. quant., Hybrid with rel. quant. targeting MTox700 + biomarkers
Instrument configuration/method	6	Polar—HILIC LC–MS (pos & neg ion modes) OR HILIC LC–MS (pos ion mode), Lipid—C18 OR C8 LC–MS (pos & neg ion modes)
Acquisition order	6	M & f separated & randomised, m & f separated & block-randomised, All samples randomised, All samples block-randomised
**OORF Data Acquisition and Processing Reporting Module: processing**		
Centroiding, baseline correction and noise reduction	6	MultiQuant, Centroiding
Data reduction	6	MultiQuant, manual inspection, Compound Discoverer, LipidSearch, MetaboScape, XCMS
Feature intensity drift and/or batch correction	6	Within-batch ultrapool normalisation, Intrastudy QC fit, Linear modelling
Identification and removal ("filtering") of features	6	Missing value filtering, dilution series correlation, process blank filtering, QC-RSD filtering, xenobiotic filtering, binning metabolite features, void volume filtering, minimum fraction filtering, sample/QC RSD
Identification and removal ("filtering") of outlying samples	6	Technical reasoning, PCA visual inspection, PCA DModX, PCA Hotelling T2, missing value filtering
Normalisation	5	Within-batch ultrapool normalisation, EigenMS, probabilistic quotient normalisation, unit normalisation, factor-level averaging, fold change relative to control
Missing value imputation	5	k-nearest neighbours, gap filling, half minimum value, NIPALS
Normality testing, scaling and/or transformations	6	Log transform, unit-variance scaling, generalized log transform
Processing methods for metabolite annotation	5	Commercial standards, public databases, theoretical *m/z*, LipidSearch, peakPanther, MetaboScape, Compound Discoverer
**OORF Data Acquisition and Processing Reporting Module: QA/QC practices**		
Intrastudy QC precision report	6	Median RSD of intrastudy QCs, Median RSD of intralaboratory QCs, PCA scores plot of QC and biological samples
**OORF Data Analysis Reporting Module: Multivariate analysis**		
Unsupervised	5	HCA, Correlation, PCA, Bootstrap PCA, Consensus PCA
Supervised	5	HCA, PLSDA, OPLSDA, LDA, SUS plots, Correlation, Bootstrapping
**Draft OORF Chemical Grouping Application Reporting Module**		
Grouping results	5	Bioactivity-based grouping, Plausible toxicological interpretation

When described at a mid-level (‘OORF reporting element’), the approaches used were largely consistent across partners (‘Used by no. of partners’). In contrast, low-level method descriptions (‘Range of methods reported’) show a wide diversity across partners. Key: m—male, f—female, pos—positive, neg—negative

A high consistency of methods is also evident from the *mid-level method descriptions* (specifically the ‘OORF reporting elements’, Table 3), with almost every process used by all 6 partners. Only a few mid-level methods (specifically ‘Normalisation’ and ‘Missing value imputation’) were less consistent, but this was due to individual, experienced ring-trial partners deliberately only applying some processing steps if warranted by their data and/or based on the other steps they applied. The high consistency of approaches used at the mid-level suggests that the OECD Omics Reporting Framework guidance document (Harrill et al. 2021), which was originally developed to guide data submitters on how to *report* ‘omics studies in a standardised manner, could also help to promote best practice and standardisation in the *use* of ‘omics approaches by indicating to metabolomics laboratories what types of data acquisition, processing and quality assessment steps should be considered, without being overly prescriptive about how individual elements should be implemented.

For the *low-level method descriptions* (Table 3 ‘Range of methods reported’, and Table S6 in Online Resource 2), there is considerably less consistency, with partners each using different approaches and/or software to achieve the same aims. For example, all partners applied mid-level method ‘Identification and removal (“filtering”) of features’, but a total of 9 different (low level) approaches were used to implement it, with some partners applying more than one approach. These observations, considering that five partners successfully grouped the eight test substances, are particularly informative, and confirm with high certainty that some variation in metabolomics approaches is *not* detrimental to achieving consistent chemical grouping. We therefore propose the mid-level method description as the minimum required to meet emerging best practices for bioactivity-based grouping using metabolomics data.

With only a single partner not reporting the correct grouping, it was not possible to reliably determine if any particular data acquisition or processing steps contributed to the underlying causes of this variation. However, what is clear from this ring trial is the importance of ‘omics data quality (assessed here using quantitative intrastudy QC measurements and visualisations of technical vs. biological metabolic variation (Viant et al. 2019)), with a relationship observed between LC–MS analytical reproducibility and accuracy of predicted group membership. For the case of regulatory submissions of metabolomics data for chemical grouping, this suggests the assessment of a data package by a regulator should include a detailed review of the “Demonstration of quality of mass spectrometry metabolomics analysis” within the OECD Omics Reporting Framework (Harrill et al. 2021) and ensuring that the analytical and computational methods adhere to best practice defined at a *mid level* of method description.

## Conclusions

Through a blinded multi-laboratory ring trial, with plasma samples derived from a single animal study, we have demonstrated both a high reproducibility and accuracy of grouping chemicals when based upon the bioactivity similarities calculated using LC–MS metabolomics data. Five of six ring-trial partners, whose metabolomics datasets passed quality control, correctly identified the grouping of eight test substances into three categories, for both male and female rats. Strikingly, this was achieved even though a range of metabolomics approaches using different analytical platforms and data evaluation strategies were used, clearly evidencing the effectiveness and robustness of this technology. Based on a detailed comparison of the data processing workflows, high- and mid-level descriptors of the methods highlighted that ring-trial partners applied similar approaches, yet low-level method descriptors revealed a wide discrepancy. We conclude that some heterogeneity in metabolomics approaches is *not* detrimental to achieving consistent chemical grouping. Furthermore, the importance of conducting quality assessments of processed metabolomics data was markedly highlighted. Through assessing intrastudy QC samples, both quantitatively and visually, the sixth ring-trial partner identified unusually high technical variation in their dataset and was not able to group the test substances. Taken together, these findings suggest the assessment of a metabolomics data package by a chemical regulator should give significant weight to ensuring high data quality was achieved from following best practice guidelines defined at a mid-level method description. We conclude that clearer international guidance is needed for metabolomics QC acceptance criteria in regulatory toxicology. It is noteworthy that existing international guidance for *reporting* ‘omics studies in regulatory toxicology (Harrill et al. 2021) already helps to promote standardised practices (i.e., by data generators following high- and mid-level method descriptors), although that guidance was not intended for this purpose and does not replace the need for metabolomics QC acceptance criteria. A particular challenge was identified in the ring trial by all partners, how to analyze test substances causing weak (or no) perturbations to the metabolome. We conclude that international guidance should also be developed on setting a threshold for ‘responder’ vs. ‘non-responder’, in a methodology agnostic way. Additionally, best practice for chemical grouping using metabolomics data will need to go beyond evidence provided by bioactivity-based methods alone for the category justification (as reported here), and incorporate plausible toxicological interpretations of the observed molecular effects. Such work is currently underway in the MATCHING study, first requiring the annotation and/or identification of features according to international standards (Sumner et al. 2007). Overall, however, the work reported here demonstrates the reliability of metabolomics for chemical grouping and contributes significantly towards the uptake of metabolomics for regulatory applications as well as working towards best practices.

### Supplementary Information

Below is the link to the electronic supplementary material.

Supplementary file1 (PDF 1267 KB)

Supplementary file2 (PDF 98 KB)

## Data Availability

The experimental metabolomics data generated during the current study are available in the MetaboLights repository under the identifier MTBLS8274.
