# Supplementary material for: Demonstrating the reliability of in vivo metabolomics based chemical grouping: towards best practice
Source: Arch Toxicol. 2024 Feb 18;98(4):1111–23. doi: 10.1007/s00204-024-03680-y (PMC10944399; doi:10.1007/s00204-024-03680-y)
Supplement: Supplementary file 1 — Supplementary file1 (PDF 1267 KB) [file 204_2024_3680_MOESM1_ESM.pdf]

Demonstrating the reliability of in vivo metabolomics based chemical grouping: Towards best practice

M. R. Viant[1], E. Amstalden[2], T. Athersuch[3], M. Bouhifd[4], S. Camuzeaux[5], D. M. Crizer[6], P. Driemert[7], T. Ebbels[3], D. Ekman[8], B. Flick[9,+], V. Giri[9], M. Gómez-Romero[5], V. Haake[7], M. Herold[7], A. Kende[10], F. Lai[10], P.E.G. Leonards[2], P. P. Lim[10], G. R. Lloyd[1], J. Mosley[8], C. Namini[8], J. R. Rice[6], S. Romano[8], C. Sands[5], M. J. Smith[1], T. Sobanski[4], A. D. Southam[1], L. Swindale[10], B. van Ravenzwaay[9,++], T. Walk[7], R. J. M. Weber[1], F. M. Zickgraf[9] and H. Kamp[7]

[1]Phenome Centre Birmingham, University of Birmingham, Edgbaston, Birmingham, B15 2TT, United Kingdom

[2]Amsterdam Institute for Life and Environment (A-LIFE), Vrije Universiteit Amsterdam, De Boelelaan 1085, 1081 HV, Amsterdam, The Netherlands

[3]Division of Systems Medicine, Department of Metabolism, Digestion & Reproduction, Imperial College London, Hammersmith Hospital, Du Cane Road, London W12 0NN, United Kingdom

[4]European Chemicals Agency, Telakkakatu 6, FI-00121, Helsinki, Finland

[5]National Phenome Centre, Department of Metabolism, Digestion and Reproduction, Imperial College London, London W12 0NN, United Kingdom

[6]Division of Translational Toxicology, National Institute of Environmental Health Sciences, Research Triangle Park, NC 27709, USA

[7]BASF Metabolome Solutions GmbH, Tegeler Weg 33, 10589 Berlin, Germany

[8]Center for Environmental Measurement and Modeling, Environmental Protection Agency, Athens, GA 30605, USA

[9]BASF SE, Carl-Bosch-Str 38, 67056 Ludwigshafen, Germany

[10]Syngenta, Jealott's Hill International Research Centre, Bracknell, RG42 6EY, United Kingdom

[+] Present address: NUVISAN ICB GmbH, Toxicology, 13353 Berlin, Germany

[++] Present address: Environmental Sciences Consulting, 67122 Altrip, Germany

* Corresponding author (Mark R. Viant)

E-mail: m.viant@bham.ac.uk

Address: Phenome Centre Birmingham, University of Birmingham, Edgbaston, Birmingham, B15 2TT, United Kingdom

ORCID: https://orcid.org/0000-0001-5898-4119

SUPPLEMENTARY MATERIALS - Online Resource 1

MATERIALS AND METHODS

Table S1. Summary of the organisations involved in the ring-trial, their role(s), and whether they were blinded to the identities of the test substances.

Purpose	Organisation	Blinded?
Test substance selection	• BASF SE<br>• European Chemicals Agency (ECHA)	No
Standard BASF evaluation of quality of plasma samples to ensure chemical grouping is as anticipated (including LC-MS, GC-MS and hormone profiling)	• BASF SE<br>• BASF Metabolome Solutions (BMS) acquired the metabolomics data	BASF SE (Kamp) - No<br>BASF SE (Zickgraf) - Yes<br>BMS - Yes
Acquire and analyse metabolomics data as a ring-trial partner	• BASF Metabolome Solutions (BMS)<br>• US Environmental Protection Agency (EPA)<br>• National Phenome Centre, Imperial College London (ICL)<br>• Syngenta (Syn)<br>• Phenome Centre Birmingham, University of Birmingham (UoB)<br>• Vrije Universiteit Amsterdam (VU)	Yes

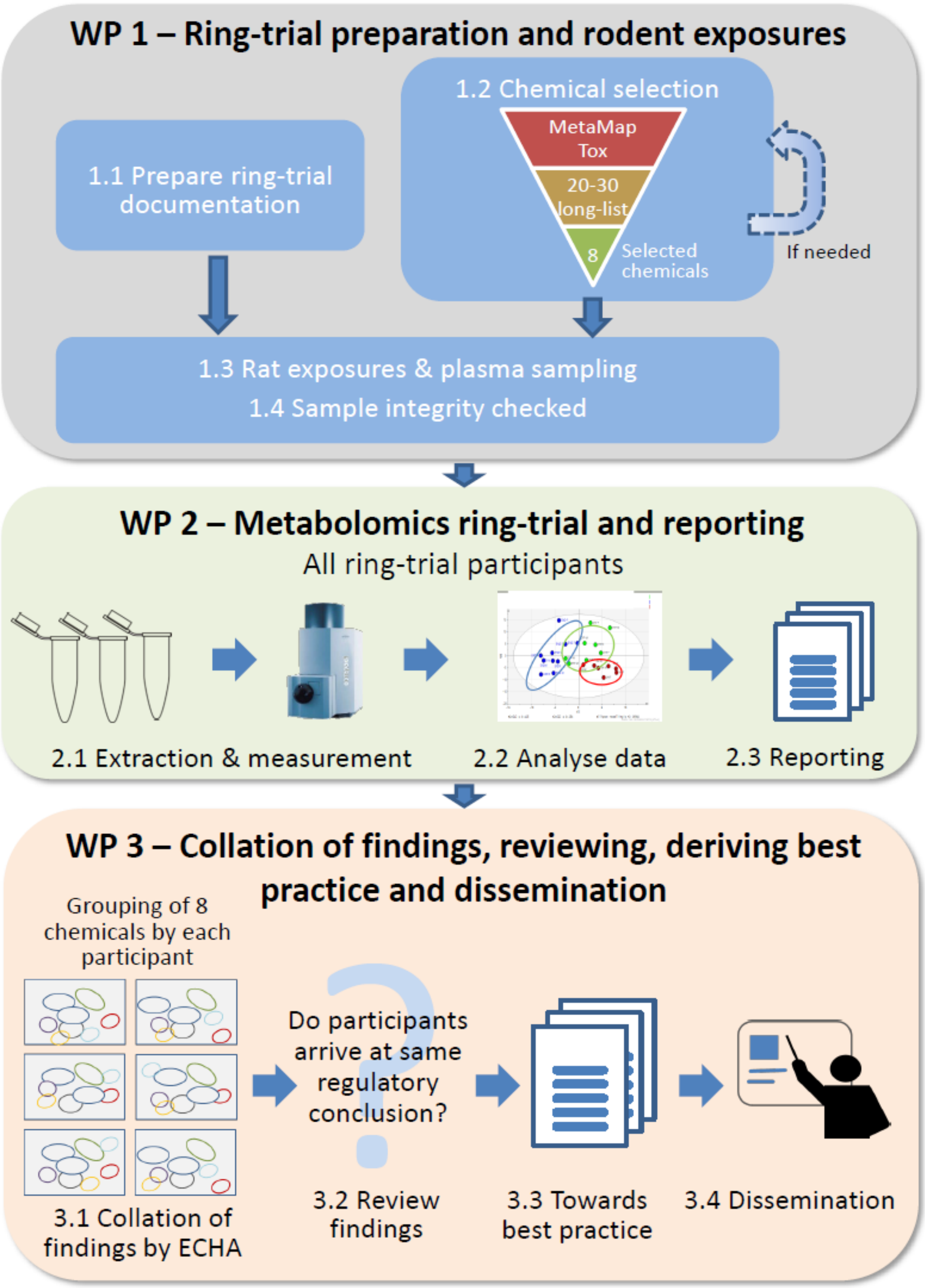

Figure S1. Overall structure of the MATCHING ring-trial study, comprising three work packages (WP).

Table S2. List of the 180 rat plasma samples investigated in the ring-trial. *Due to limited commercial availability, substance TS6 was replaced by the backup substance TS9.

Code*	Test substance	Dose level	Replicate	Animal no.	
				Males	Females
TS0	Control	0	01-10	001 to 010	091 to 100
TS1	Wy 14643	LD	01-05	011 to 015	096 to 105
		HD	01-05	016 to 020	101 to 110
TS2	4-Chloro-3-nitroaniline	LD	01-05	021 to 025	106 to 115
		HD	01-05	026 to 030	111 to 120
TS3	17α-Methyl-testosterone	LD	01-05	031 to 035	116 to 125
		HD	01-05	036 to 040	121 to 130
TS4	Trenbolone	LD	01-05	041 to 045	126 to 135
		HD	01-05	046 to 050	131 to 140
TS5	Aniline	LD	01-05	051 to 055	136 to 145
		HD	01-05	056 to 060	141 to 150
TS7	Dichlorprop-p	LD	01-05	061 to 065	146 to 155
		HD	01-05	066 to 070	151 to 160
TS8	2-Chloroaniline	LD	01-05	071 to 075	156 to 165
		HD	01-05	076 to 080	161 to 170
TS9	Fenofibrate	LD	01-05	081 to 085	166 to 175
		HD	01-05	086 to 090	171 to 180

Table S3. Naming convention used for the rat plasma samples: RP[01-08]_TS[0-9]_DG[0-2]_[m|f]_rep[01-10]_[001-180].

**Component**	**Description**
RP[01-08]	Ring-trial partner identifier (01 to 08, randomly assigned to each ring-trial partner by ECHA; partner identities were blinded in the ring-trial). Note that two of the sample sets were not investigated by the ring-trial partners.
TS[0-9]	Test substance (0, 1, 2, 3, 4, 5, (*no 6*), 7, 8, 9), where TS0 = control samples
DG[0-2]	Dose group: 0 = control group, 1 = low, 2 = high
[m|f]	Sex of animal: m for male, f for female
rep[01-10]	Biological replicate within each group, from 01 to 05 for treated groups, 01 to 10 for control group
[001-180]	Animal number, unique to each animal in this study

Section S1. Animal exposures and plasma sampling by BASF SE

Ethics Statement

The *in vivo* studies were approved by the BASF Animal Welfare Body and performed according to the German Animal Welfare Act and EU Directive 2010/63, with the permission of the local authority, the Landesuntersuchungsamt Rheinland-Pfalz (permission number 23 177-07/G 18-3-098). The laboratory is AAALAC (Association for Assessment and Accreditation of Laboratory Animal Care International) certified.

Animal Maintenance and Administration

For this study, Wistar rats (Crl:WI(Han)) were obtained from Charles River Laboratories, Sulzfeld, Germany. At the beginning of the treatment the ages of the animals were 70 +/- 5 days. The animals were housed together (5 animals per cage) in H-Temp polysulfonate cages (type 2000P, floor area about 2065 cm^2). Dust free wooden bedding was used, and wooden gnawing blocks (LIGNOCEL® block large) were added for environmental enrichment. The animals were accommodated in fully air-conditioned rooms with a temperature range of 20-24°C and relative humidity of 45-65%. The day/night cycle was 12 hours. The diet and drinking water were available ad libitum (except before blood sampling) and were regularly assayed for chemical contaminants and the presence of micro-organisms. There were 5 animals per sex per treatment group and 10 animals per sex in the control group. The animals were treated with each of the eight test compounds orally, via the diet or gavage, as indicated in Table 1, daily for 28 days. The test substances were mixed into the powdered diet or (OECD 2008) prepared as solution in either corn oil, deionised water, or deionised water containing 0.5% carboxymethylcellulose. The volume to be administered was 5 mL/kg body weight in the case of corn oil and deionised water. The control animals received the standard diet, only.

Dose levels

The dose levels were selected based on previously conducted 28-day repeated dose studies. High dose levels were chosen to induce clear effects without causing suffering to the animals and not exceeding the maximum tolerated dose for a 28-day study. The dose levels used are shown in Table 1.

Animal examinations and sampling

Although no regulatory test guideline exists for this type of study, for parts of the study, i.e., clinical examinations, clinical chemistry and sampling for histopathology, reference was made to the OECD Guidelines for Testing of Chemicals; Method No. 407: Repeated Dose 28-day Oral Toxicity Study in Rodents. Briefly, the following parameters were determined: mortality, clinical signs of toxicity, body weight, food consumption, haematology, organ weights, and macroscopic pathology. Tissues for potential histopathological examinations were fixed and stored. On study day 21, blood was taken from non-fasted animals by puncturing the vena saphena without anaesthesia (30 µL blood were diluted in 150 µL NaCl (1.5%)) for measuring haematological parameters.

Metabolomics blood sampling and aliquoting

Blood samples were taken from fasted animals by puncturing the retrobulbar venous plexus on study days 7 and 14 for all test groups under isoflurane anaesthesia, and on study day 28 after decapitation under isoflurane anaesthesia. On study day 7 and 14, 1 mL of blood was collected from each animal in Eppendorf tubes with EDTA as anticoagulant (10 µL of a 10% solution). On study day 28, as much blood as possible was collected from each animal in 13 mL Sarstedt tubes, and per 1 mL of blood 10

µL of a 10% solution of EDTA was added. The samples were centrifuged, and the plasma was separated. The blood samples taken on study days 7 and 14 were stored as backup without further aliquoting (these samples were not analysed further). Next, for each animal, plasma samples from study day 28 were subdivided into 7 aliquots of 160 µL each for the external ring-trial partners, as well as 1 aliquot of 750 µL for the internal partner (BMS) who took part in the ring trial and conducted the sample quality-control to ensure that the treatments resulted in clear metabolic changes from controls. The preparation of the plasma samples was done under cooling.

All plasma samples were stored in original Eppendorf tubes covered with a N_2 atmosphere at –80°C. The samples were sent on dry ice to BASF Metabolome Solutions GmbH (BMS), Berlin, Germany. To each partner, BMS distributed the plasma samples, as well as (a) standard reference metabolite mixes (polar and lipid) provided by Syngenta, and (b) rat plasma long-term-reference material and pre-extracted rat plasma long-term-reference material provided by BMS, which could be used for a detailed inter-laboratory comparison, if needed.

Each partner received a set of 180 samples, comprising 160 treated samples (10 animals per group (5 male, 5 female) x 8 substances x 2 concentrations x 1 time point), and 20 control samples (10 animals per sex), listed in Table S2. Each sample label contained the ring trial partner number, the substance code, dose group, rat sex, biological replicate and animal number (e.g., RP02_TS1_DG2_m_rep02_017), as defined in Table S3.

References

OECD (2008) Test No. 407: Repeated Dose 28-day Oral Toxicity Study in Rodents, Section 4. OECD Publishing, Paris. https://doi.org/10.1787/9789264070684-en

Table S4. Summary of analytical approaches used by ring-trial partners.

Ring-trial partner	Extraction solvents	LC-MS instrumentation	LC-MS methods
BMS	MeOH:DCM:water: toluene (93:47:16,5:1, v/v)	Shimazu MPX System, AB Sciex QTrap 6500+	Targeted analysis<br>Polar: HILIC (pos & neg)<br>Non-polar: C18 (pos & neg)
EPA	Polar: MeOH:ACN<br>Non-polar: IPA	Thermo Vanquish UHPLC, Thermo Q Exactive	Untargeted analysis<br>Polar: HILIC (pos & neg)<br>Non-polar: C18 (pos & neg)
ICL	Polar: ACN<br>Non-polar: IPA	Waters Acquity UPLC, Waters QToF Xevo G2-S	Hybrid analysis<br>Polar: HILIC (pos)<br>Non-polar: C8 (pos & neg)
Syn	Polar: MeOH:ACN<br>Non-polar: IPA	Polar: Thermo Vanquish UHPLC, Thermo Exploris 240<br>Non-polar: Thermo Vanquish UHPLC, Thermo Q Exactive Plus	Hybrid analysis, targeting MTox700+ biomarkers<br>Polar: HILIC (pos & neg)<br>Non-polar: C18 (pos & neg)
UoB	Polar: MeOH:ACN<br>Non-polar: IPA	Thermo Ultimate 3000 UHPLC, Thermo Q Exactive Focus	Hybrid analysis, targeting MTox700+ biomarkers<br>Polar: HILIC (pos & neg)<br>Non-polar: C18 (pos & neg)
VU	Polar: MeOH:ACN<br>Non-polar: IPA	Agilent UHPLC, Bruker Compact QToF	Untargeted analysis<br>Polar: HILIC (pos & neg)<br>Non-polar: C18 (pos & neg)

Section S2. Acquisition and processing of LC-MS metabolomics data

This section describes the methods for sample extraction, acquisition, and processing of the LC-MS metabolomics data, by each partner. Organisation names are used in this section as the methods do not reveal the identities of the partners' results.

BASF Metabolome Solutions (BMS)

Sample processing for mass spectrometry-based metabolite profiling of blood plasma was performed according to a standardised protocol (Murali et al. 2023). Targeted metabolites were measured from 60 µL rat plasma extracted by adding 1500 µL extraction buffer (methanol, dichloromethane, water and toluene (93:47:16,5:1, v/v) buffered with ammonium acetate) using a ball mill (Bead Ruptor Biolab). Internal standards (see details below) were added to the extraction mixture to enable reproducibility analysis. After centrifugation, (11290-g, 10min at 12°C), a 100 µL aliquot of the extract was taken for LC-MS/MS (the standard protocol was used where the remaining extract was available for use in GC-MS analysis).

For LC-MS/MS mass spectrometry analysis, 2.5 µL of the extract were injected each for reversed-phase and hydrophilic interaction liquid chromatography followed by MS/MS detection (AB Sciex QTrap API 6500+ triple quadrupole) with both positive and negative ionization mode. As MS acquisition mode(s) scheduled "MultipleReactionMonitoring" (MRM) with parallel polarity switching (positive/negative ion analysis) was applied. To enable fast analysis, an AB-Sciex MpX-System was used for chromatography. It was combining two Shimadzu 20 LC-systems in one larger setup enabling fast switching between two different LC-streams. LC-stream-1 was based on reverse-phase high performance liquid chromatography (Ascentis Express C18, 50 mm x 2.1 mm, 2.7 µm Supelco) for optimal separation of lipid metabolites. Gradient elution was performed with (A) water/methanol/0.1 M ammonium formate (1:1:0.02 w/w) and (B) methyl-tert-butylether/2-propanol/methanol/0.1 M ammonium formate (2:1:0.5:0.035 w/w) with 0.5% (w%) formic acid with the following profile: 0 min 100% A, 0.5 min 75% A (25% B), 5.9 min 10% A (90% B) at 40°C and a flow rate of 600 µL/min. LC-stream-2 was applying hydrophilic interaction liquid chromatography (ZIC - HILIC, 2.1 x 100 mm, 3.5 µm, Supelco) for optimal analysis of polar metabolites. Here, gradient elution was performed with (C) acetonitrile/water (99:1, v/v) and 0.2 (v%) acetic acid and (D) 0.007 M ammonium acetate with 0.2 (v%) acetic acid with the following profile: 0 min 100% C, 5 min 10% C (90% D), at 40°C and a flow rate of 600 µL/min.

To avoid any storage effects after extraction, the full set of 90 study samples per gender plus quality-control samples was analysed in five blocks with handling being optimised to fit into a 24h turnaround routine. Throughout the entire processing, samples were tracked using a LIMS system with barcoded labels. Per gender, the assignment of the samples into the different blocks and the order of analysis were pre-determined by computer-based randomization using a BASF proprietary script based on the following logic: in the first step, samples from the different test-groups were distributed equally across the five analysis blocks. After sample assignment to blocks, they were shuffled within these blocks in 10 iterations. Finally, the Shannon entropy was used to determine the most randomised design from those 10 iterations. This way, it was ensured that the different groups were completely randomised across the entire analysis. Five ultrapool reference samples (see below) were distributed throughout each block at fixed positions (see below for details). The first extraction step was the addition of the extraction buffer to quench any remaining enzymatic activity. The extraction buffer also contained internal standards (isotope-labelled metabolites that were used to evaluate any potential systematic

sample volume differences during the handling procedure. For extraction, samples were handled separately for each sex in batches with each extraction batch comprising 5 ultrapool samples, linearity samples (ultrapool at 12.5, 25, 50 and 150% concentration) plus study samples. The sample order was maintained throughout the entire process.

To evaluate instrument performance, a solvent sample followed by two standard mix samples and another solvent sample were run prior. When the instrument passed the QC criteria, the analysis of the study samples was started. All biological samples were analysed once in a randomised analytical sequence design to avoid artificial results with respect to analytical shifts. For LC-MS/MS profiling, data were normalised to the median of reference samples (so called ultrapool samples) to account for inter- and intra-instrumental variation. The rat plasma ultrapool was generated previously by pooling plasma from a large number of male and female rats in a dedicated effort to generate a large lot of reference material to serve as consistent reference material in all BASF rat plasma studies. This ultrapool was aliquoted, freeze-dried and stored at -80°C until use.

LC-MS/MS chromatograms were first processed using the commercial software MultiQuant obtained from the MS instrument supplier (Sciex, version 3.0.3). The processing method had been generated based on the independent method development process that was done outside the scope of this study. In short, during method development, ultrapool samples were analysed to determine key peak parameters such as typical peak shape, mass transitions for MS/MS quantification, retention times and retention time windows. These pre-determined values then built the foundation for MultiQuant processing in this study. The processed chromatograms were manually inspected by bioanalytical experts for any obvious integration errors or general technical problems. Obvious misintegrations were manually corrected at analyte level across all samples to avoid any between sample bias.

In our targeted approach, a pre-defined set of metabolites are identified by their analytical parameters: polarity (lipid vs polar), MRM transition (*m/z* ratios), and retention time. To confirm the identity of a metabolite, samples were spiked with the pure metabolite during method development whenever possible. When metabolites were not commercially available, fragmentation patterns and library matching were used to determine the most likely identity of those metabolites. Using a normalisation strategy based on rat plasma ultrapool according to patent WO2007012643A1 (Walk et al. 2011), ultrapool-ratio values were generated. These built the foundation for all subsequent statistical analyses of the study samples.

The targeted method used in this study was strictly based on features that were also identified in untreated ultrapool reference samples. Therefore, no removal of features from dosed substances was required (disclaimer: this assumed that none of the dosed substances was also naturally occurring in untreated control animals at some lower level). During the development of the targeted method (outside scope of this report) used in this study, a concentration series for ultrapool samples (dilution or re-dissolving the lyophilised material in a smaller volume to obtain concentrations >100%) was evaluated to determine the linear range and quality of all detected analytes. Based on that the method was set up.

For this particular study, to confirm that analytes were in the instrument's linear range, an ultrapool concentration series (0, 12.5, 25, 50, 100 and 150% ultrapool concentration) was analysed.

All targeted metabolites that were used in the subsequent analyses passed the following quality criteria that were assessed in the analysis of the ultrapool concentration series:

1. data coverage of ≥ 80% for the ultrapool samples (100% concentration) to ensure robust detection
2. concentration linearity analysis showed an r^2 correlation value of ≥ 0.64 as indicator for the linearity of the response for the particular analyte
3. blank contribution < 40% (blank signal compared to ultrapool which translates into an ultrapool vs blank ratio of ≥ 2.5) to remove noisy analytes
4. ultrapool relative standard deviation (calculated based on log-scale as rsd-down to account for skewness of normalised values) was < 0.6 to show reproducibility.

Of the 323 analytes obtained from the chromatograms, 271 met all the criteria listed above and were therefore included as semi-quantitative analytes in subsequent analyses. The remaining 52 analytes were considered as qualitative only and therefore were not evaluated further in the subsequent analyses reported here.

References

Murali A, Giri V, Zickgraf FM, et al (2023) Connecting Gut Microbial Diversity with Plasma Metabolome and Fecal Bile Acid Changes Induced by the Antibiotics Tobramycin and Colistin Sulfate. Chem Res Toxicol 36:598–616. https://doi.org/10.1021/acs.chemrestox.2c00316

Walk TB, Looser R, Bethan B, et al (2011) System and Method for Analyzing Sample Using Chromatography/mass Spectrometry. JP 2011203262 A

US Environmental Protection Agency (EPA)

Sample Extraction. 180 rat plasma samples were assigned randomly to one of four batches. All plasma samples within a batch were thawed on wet ice, vortexed (5 s) then centrifuged (20,000-g, 4 °C, 10 min). Then, each sample was aliquoted 3 x 50 µL into 2 mL Eppendorf tubes and re-frozen at -80 °C. Plasma samples were re-randomised across 3 batches for each of the C18 Lipids and HILIC UHPLC-MS assays. For each batch per assay, 50 µL plasma samples and empty vials for process blanks (n=3) were thawed on ice. Each sample was vortexed for 5 s, and 150 µL of ice-cold isopropanol (Optima LC-MS grade; Fisher Scientific, Waltham, MA, USA) was added for the C18 Lipids UHPLC-MS assay, whereas 150 µL of ice-cold 50:50 methanol:acetonitrile (Optima LC-MS grade; Fisher Scientific, Waltham, MA, USA) was added for the HILIC UHPLC-MS assay. Extracts were vortexed (15 s) then centrifuged (20,000-g, 4 °C, 20 min). 50 µL were transferred to a pre-wetted 0.2 µm Captiva 96-well plate (Agilent, Santa Clara, CA, USA), centrifuged into a Microliter 96-well plate with glass vial inserts (DWK Life Sciences, Millville, NJ, USA) and kept at 4 °C for LC-MS analysis. An additional 20 µL were transferred to a large (6 mL) glass vial on ice to form an intrastudy-QC (isQC) pool. While all batches were being prepared across 2 days, the first isQC pool was kept at 4 °C, and the second isQC pool was kept on ice only. The final isQC pool (containing all 180 study samples) was vortexed for 30 s, transferred in multiple 50 µL aliquots to a pre-wetted 0.2 µm Captiva 96-well plate (Agilent, Santa Clara, CA, USA), centrifuged into a Microliter 96-well plate with glass vial inserts (DWK Life Sciences, Millville, NJ, USA) and kept at 4 °C for LC-MS analysis.

UHPLC-MS metabolomics. *Lipids*. Plasma extracts were spiked with an internal standard (IS) mixture made from the SPLASH Lipidomix standard mixture that included d7-PC(15:0/18:1), d7-PE (15:0/18:1), d7-PG(15:0/18:1), d7-PI(15:0/18:1), d7-PS-(15:0/18:1), d7-LPC(18:1), d7-LPE(18:1), d7-TG(15:0/18:1/15:0), d7-DG(15:0/18:1), d7-MG(18:1), d9-SM-(d18:1/18:1), d7-CE(18:1), and d7-cholesterol (Avanti Polar Lipids, Alabaster, AL, USA), Cer(d18:1/12:0) (Avanti Polar Lipids), and d27-

myristic acid (Sigma-Aldrich, St. Louis, MO, USA). The spiked plasma extracts were profiled in a tandem-LC configuration for positive (+ESI) and negative (-ESI) modes, respectively, using a Vanquish Flex ultra-high performance liquid chromatography (UHPLC) system coupled to a Q-Exactive orbitrap mass spectrometer (Thermo Fisher Scientific, Waltham, MA, USA) via a heated electrospray ionization source (HESI-II). All 180 samples were randomised across 6 analytical batches. IsQC pool aliquots were injected for column conditioning (n=10 for batch 3; n=20 for all other batches) immediately before the analytical batch at the beginning (n=2), end (n=1), and after every 8 samples of the run. Process blanks (n=3 total) were injected at the beginning, middle and end of the run. Study samples, process blanks and intrastudy QC samples (2 µL) were injected onto dual Hypersil Gold C18 columns (100 mm × 2.1 mm × 1.9 µm; Thermo Fisher Scientific) that were maintained at 55 °C and operated at a flow rate of 400 µL/min. The mobile phases used for analysis consisted of (A) 60% acetonitrile/water with 10 mM ammonium formate and 0.1% formic acid (v/v) and (B) 85.5% propan-2-ol/9.5% acetonitrile/5% water with 10 mM ammonium formate and 0.1% formic acid (v/v). All solvents were Optima LC-MS grade (Fisher Scientific). The following gradient was applied for lipid separation: t=0.0 min, 20% B; t=1.6, 20% B; t=9.4, 100% B; t=10.6, 100% B; t=12.6, 20% B; t=15.0, 20% B, all changes were linear with curve = 5. The sample vials were maintained on a cold rack at 10 °C during the injection. The following parameters were used during the full scan MS^1 profile analysis in positive mode: auxiliary gas heater temperature, 450 °C; sheath gas, 48; auxiliary gas, 15; sweep gas, 0; capillary temperature, 380 °C; and source voltage, 3.2 kV. In negative mode, parameters were changed to the following: source voltage, 2.7 kV. The scan range was from 150 to 2000 *m/z* and the resolution was set at 70,000. The automatic gain control target was set at 3×10^6 and maximum injection time was 200 ms.

UHPLC-MS metabolomics. *HILIC*. Plasma extracts were spiked with the QRESS IS mixture (MSK-QReSS-KIT; Cambridge Isotope laboratories, Tewksbury, MA, USA). The spiked plasma extracts were profiled using a Vanquish Flex ultra-high performance liquid chromatography (UHPLC) system coupled to a Q-Exactive orbitrap mass spectrometer (Thermo Fisher Scientific, Waltham, MA, USA) via HESI-II for +ESI and -ESI modes. All 180 samples were randomised across 3 analytical batches per ESI mode. IsQC pool aliquots were injected for column conditioning (n=20) immediately before the analytical batch at the beginning (n=2), end (n=1), and after every 9 samples of the run. Process blanks (n=2 total) were injected at the beginning and end of the run. Study samples, process blanks and isQC pool aliquots (2 µL) were injected onto Accucore 150 Amide HILIC columns (100 mm × 2.1 mm × 2.6 µm; Thermo Fisher Scientific) that were maintained at 35 °C and operated at a flow rate of 500 µL/min. The mobile phases used for analysis consisted of (A) 95% acetonitrile/water with 10 mM ammonium formate and 0.1% formic acid (v/v) and (B) 50% acetonitrile/water with 10 mM ammonium formate and 0.1% formic acid (v/v). All solvents were Optima LC-MS grade (Fisher Scientific). The following gradient was applied for polar metabolite separation: t=0.0 min, 1% B; t=2.1, 1% B; t=4.1, 15% B; t=7.1, 50% B; t=10.1, 95% B; t=11.0, 95% B; t=11.5, 1%B; t=15.0, 1% B, all changes were linear with curve = 5. The sample vials were maintained on a cold rack at 4 °C during the injection. The following parameters were used during the full scan MS^1 profile analysis in positive mode: auxiliary gas heater temperature, 440 °C; sheath gas, 55; auxiliary gas, 14; sweep gas, 4; capillary temperature, 380 °C; and source voltage, 3.2 kV. In negative mode, parameters were changed to the following: source voltage, 2.7 kV. The scan range was from 70 to 1050 *m/z* and the resolution was set at 70,000. The automatic gain control target was set at 3×10^6 and maximum injection time was 200 ms.

Data processing. The raw data files were converted to open-source format (.mzML) using the "MSConvert" program from ProteoWizard (Chambers et al. 2012), and processed using XCMS (v. 3.2.0;

Smith et al. 2006) and CAMERA (v. 1.36.0; Kuhl et al. 2012) in the R environment (v 3.5.1), as previously described (Mosley et al. 2018). The feature lists from each mode were curated with the LUMA package in R (v. 2.0.1; Evich et al. 2020). Specifically, lists were trimmed by removing features contained within the void volume, removing those features identified as coming from background contaminants (mean intensity in process blank samples higher than 10% (for polar metabolites) or 1/3 (for lipids) of the mean intensity in the isQC samples), retaining only those that were present in at least 80% of the isQC samples (both lipids and polar metabolites) and exhibited a percent coefficient of variation of <35% in the isQC samples (lipids only), and removing those features that were not present in at least 80% of one exposure class, as these were considered not to be reliable biomarkers. At this point, we defined the remaining features as endogenous if their mean intensity was greater than 1×10^5 in the control samples, and exogenous if not. (Exogenous features were not included in the subsequent analysis). Metabolite intensities were normalised to unit total integrated intensity for each ion mode individually. The resulting endogenous metabolite lists from both positive and negative ionization modes were combined into a single dataset per assay. These steps resulted in a lipid dataset with a total of 1830 features (1195 positive and 635 negative features), and a polar metabolite dataset with a total of 1437 features (704 positive and 733 negative features).

QA/QC. Real-time checks of signal response for select IS features were performed to flag sample outliers, e.g., due to injection errors. Failures in individual samples or batches triggered a re-analysis in a later batch. Once all data was acquired and passed the real-time checks, the main sources of analytical variability, or drift, were assessed post-hoc and corrected (where necessary) to ensure the high quality of feature lists for each assay for downstream analysis. Specifically, *m/z* drift was assessed for the entire *m/z* range on select IS features across representative isQC samples from each analytical batch. If the error due to *m/z* drift exceeded 3 ppm, mass calibration was performed on the feature list using the “edgeshift” method in XCMS. Retention time (RT) drift was assessed and corrected using the “obiwarp” method in XCMS. RT drift was considered acceptable if the difference between adjusted and raw RT was within ± 2% of the total chromatographic run (15 min). Signal response (intensity) drift was assessed by calculating percent relative standard deviation (RSD) for all features across all isQC samples from raw (uncorrected) feature lists and by PCA scores plots generated from pre-processed feature lists containing all isQC and study samples, which were visually inspected both for batch effects and for tight clustering of isQC samples near the origin. If visible batch effects or poor clustering of isQC’s were observed, a correction was applied using quality control-robust spline correction (QC-RSC) (Kirwan et al. 2013) as implemented in the pmp package in R (v. 1.2.1; Jankevics et al. 2021).

References

Chambers MC, Maclean B, Burke R, et al (2012) A cross-platform toolkit for mass spectrometry and proteomics. Nat Biotechnol 30:918–920. https://doi.org/10.1038/nbt.2377

Evich MG, Melendez W, Mosley JD (2020) The LCMS-Based Untargeted Metabolomics Assistant (LUMA) R package. https://cfpub.epa.gov/si/si_public_record_report.cfm?Lab=CEMM&dirEntryId=349142

Jankevics A, Lloyd GR, Weber RJM (2021) Peak Matrix Processing and signal batch correction for metabolomics datasets. https://doi.org/doi:10.18129/B9.bioc.pmp

Kirwan JA, Broadhurst DI, Davidson RL, Viant MR (2013) Characterising and correcting batch variation in an automated direct infusion mass spectrometry (DIMS) metabolomics workflow. Anal Bioanal Chem 405:5147–5157. https://doi.org/10.1007/s00216-013-6856-7

Mosley JD, Ekman DR, Cavallin JE, Villeneuve, DL, Ankley, GT, & Collette, TW (2018). High-resolution mass spectrometry of skin mucus for monitoring physiological impacts and contaminant biotransformation products in fathead minnows exposed to wastewater effluent. Environ Toxicol Chem, 37(3), 788-796. https://doi.org/10.1002/etc.4003
Kuhl C, Tautenhahn R, Böttcher C, et al (2012) CAMERA: An Integrated Strategy for Compound Spectra Extraction and Annotation of Liquid Chromatography/Mass Spectrometry Data Sets. Anal Chem 84:283–289. https://doi.org/10.1021/ac202450g
Smith CA, Want EJ, O'Maille G, et al (2006) XCMS: Processing Mass Spectrometry Data for Metabolite Profiling Using Nonlinear Peak Alignment, Matching, and Identification. Anal Chem 78:779–787. https://doi.org/10.1021/ac051437y

National Phenome Centre, Imperial College London (ICL)

Samples were prepared and acquired by ultra-high performance liquid chromatography mass spectrometry (UHPLC-MS) using previously published analytical and quality-control (QC) procedures to generate high quality datasets (Izzi-Engbeaya et al., 2018; Lewis et al., 2016). Assays comprised a reversed-phase chromatography (RPC) using an acyl chain bonded phase (Waters Acquity UPLC BEH C8, 1.7 µm, 2.1 x 100 mm) tailored for the separation of lipophilic analytes (e.g., complex and neutral lipids) and hydrophilic interaction liquid chromatography (HILIC) using a unbonded ethylene bridge hybrid particle (Waters Acquity BEH HILIC, 1.7 µm, 2.1 x 150 mm) for separation of hydrophilic analytes (e.g., polar and charged metabolites).

For QC and pre-processing, a pooled isQC sample was prepared by combining equal parts of each study sample and acquired at 10 study sample intervals during sample analyses. An additional set of QC sample dilutions was created (100%, 80%, 60%, 40%, 20%, 10%, 1% acquired 10, 5, 3, 3, 5, 10, 10 times, respectively) and analysed at the start and end of each sample analyses for assessment of analyte response. Samples were acquired in an order randomised against key study design elements (sex, treatment group, dose group).

Samples were prepared as previously described (Lewis et al. 2016; Izzi-Engbeaya et al. 2018). In brief, 50 µL aliquots were taken from each sample, sample aliquot and isQC were further diluted 1:1 v/v with ultrapure water. Protein was removed by addition of organic solvent (diluted sample/isopropanol in 1:4 v/v ratio for lipid RPC profiling and diluted sample/acetonitrile in 1:3 v/v ratio for HILIC profiling). Mixtures of method specific authentic reference materials and internal standards were added (at dilution step for HILIC or protein precipitation step for RPC) to monitor data quality during acquisition.

Analyses were performed on ACQUITY UPLC instruments (Waters Corp., Milford, MA, USA) coupled to Xevo G2-S TOF mass spectrometers (Waters Corp., Manchester, UK) via a Z-spray electrospray ionisation (ESI) source operating in both positive and negative ion modes. Datasets consisted of lipid RPC positive and negative (Lipid RPC+ and Lipid RPC- respectively) and HILIC positive (HILIC+). Acquisition settings were as follow for lipidomics: sampling cone 25 V, source temperature 600 °C, cone gas 150 L/Hr, desolvation 1000 L/Hr, capillary voltage 2 kV (Lipid RPC+) and 1.5 kV (Lipid RPC-) and mass range 50-2000 *m/z*. For HILIC: sampling cone 20 V, source temperature 600 °C, cone gas 150 L/Hr, desolvation 1000 L/Hr, capillary voltage 1.5 kV (HILIC+) and mass range 50-1200 *m/z*. The following chromatographic conditions were applied for lipidomics: mobile phase A: water/isopropanol/acetonitrile 2:1:1, 5 mM ammonium acetate, 0.05% acetic acid, 20 µM phosphoric acid; mobile phase B: isopropanol/acetonitrile 1:1, 5 mM ammonium acetate, 0.05% acetic acid;

column temperature 55 °C; flow rate 0.6 mL/min; gradient profile t=0, 1% B; t=0.1, 1% B; t=2, 30% B; t=11.5, 90% B; t=12, 99.9% B; t=12.5, 99% B; t=13.25, 1% B; injection volume 2 µL (Lipid RPC-) and 1 µL (Lipid RPC+). For HILIC: mobile phase A: 0.1% formic acid, and 20 mM ammonium formate in water; mobile phase B: 0.1% formic acid in acetonitrile; column temperature 40 °C; flow rate 0.6 mL/min; gradient profile t=0, 95% B; t=0.1, 95% B; t=4.6, 80% B; t=5.5, 50% B; t=7.0, 50% B; t=7.1, 95% B; t=12.65, 95% B; injection volume 2 µL.

Raw data was converted to the mzML open-source format and signals below an absolute intensity threshold of 100 counts were removed using the MSConvert tool in ProteoWizard (Chambers et al. 2012). To produce profiling datasets, feature extraction was performed using XCMS (Smith et al. 2006). During the grouping step of feature extraction, sample groups were defined as blanks, control female, control male and all other samples. This allowed exclusion of peaks detected in blanks or not detected in control samples, for blank and control (exogenous) filtering.

In addition, extraction of target metabolites from the mzML files was performed using peakPantheR, an R package for targeted integration of chemical signals from LC-MS datasets (Wolfer et al. 2021). Using peakPantheR, for each UHPLC-MS dataset, empirical retention time and theoretical *m/z* values from an in-house database of metabolite/lipid annotations were integrated yielding complimentary datasets of known and pre-assigned metabolites. For these datasets, blank and exogenous filtering was performed by first estimating the limit of detection (LOD) for each metabolite using *LOD = mean(blanks) + 3 * stdev(blanks)*, then excluding any metabolites where *mean(control) < 3 * LOD*.

All datasets were pre-processed using the nPYc-Toolbox (Sands et al. 2019) including elimination of potential run-order effects and feature filtering. Only features measured with high analytical quality (RSD in isQC <30%, pooled QC dilution series Pearson correlation to dilution factor >0.7, RSD in study samples >1.1× RSD in isQC) were retained and put forward for further statistical analysis. After feature filtering, profiling datasets contained the following number of variables; HILIC+: 773; Lipid RPC-: 318; Lipid RPC+: 976 and targeted extraction datasets contained the following number of metabolites; HILIC+: 49; Lipid RPC-: 70; Lipid RPC+: 278.

References

Chambers MC, Maclean B, Burke R, et al (2012) A cross-platform toolkit for mass spectrometry and proteomics. Nat Biotechnol 30:918–920. https://doi.org/10.1038/nbt.2377

Izzi-Engbeaya C, Comninos AN, Clarke SA, et al (2018) The effects of kisspeptin on β-cell function, serum metabolites and appetite in humans. Diabetes Obes Metab 20:2800–2810. https://doi.org/10.1111/dom.13460

Lewis MR, Pearce JTM, Spagou K, et al (2016) Development and Application of Ultra-Performance Liquid Chromatography-TOF MS for Precision Large Scale Urinary Metabolic Phenotyping. Anal Chem 88:9004–9013. https://doi.org/10.1021/acs.analchem.6b01481

Sands CJ, Wolfer AM, Correia GDS, et al (2019) The nPYc-Toolbox, a Python module for the pre-processing, quality-control and analysis of metabolic profiling datasets. Bioinformatics 35:5359–5360. https://doi.org/10.1093/bioinformatics/btz566

Smith CA, Want EJ, O'Maille G, et al (2006) XCMS: Processing Mass Spectrometry Data for Metabolite Profiling Using Nonlinear Peak Alignment, Matching, and Identification. Anal Chem 78:779–787. https://doi.org/10.1021/ac051437y

Wolfer AM, D S Correia G, Sands CJ, et al (2021) peakPantheR, an R package for large-scale targeted extraction and integration of annotated metabolic features in LC–MS profiling datasets. Bioinformatics 37:4886–4888. https://doi.org/10.1093/bioinformatics/btab433

Syngenta (Syn)

Samples were analysed using liquid chromatography – high resolution mass spectrometry (LC-HRMS) applying two hybrid metabolomics methods, one for polar/midpolar (PMP) and one for lipid metabolites using HILIC and C18 phase separations, respectively, according to previously published analytical and QC procedures (Kende et al. 2023). In brief, from the plasma samples provided two, 50 µL aliquots were generated, spiked with the assay specific extraction standard and then extracted with lipid and polar/midpolar extraction solvents: 300 µL isopropyl alcohol and 150 µL acetonitrile:water (4:1), respectively. The recovery check samples of the extraction standards for each assay and each batch were generated using two, 50 µL aliquots of rat plasma (intralab QC, not study related). The samples were vortexed, sonicated, centrifuged, filtered and fortified with injection standard prior to analysis in randomised sample preparation order. An extraction blank was included in each batch for each method. The assay specific isQC samples were created from aliquots of the respective methods' extracts and re-injected at regular intervals throughout the runs. The extraction standards were used to monitor the reproducibility of the extraction and the injection standard was used for monitoring instrument performance but were not used for data corrections.

The PMP assay was run on Thermo Exploris 240 with Vanquish UHPLC and the Lipidomics assay was run on Thermo Q Exactive Plus with Vanquish UHPLC. The HILIC chromatographic method was adopted from Phenome Centre Birmingham with permission (see UoB methods, below), the MS settings were developed in-house. The lipidomics assay uses apolar reversed phase separation on C18 (Phenomenex Kinetex 100 Å, 2.6µm, 2.1mm x 150mm) with eluent A: 10 mM ammonium formate and 0.1% formic acid in 60% acetonitrile/water and eluent B: 10 mM ammonium formate and 0.1% formic acid in 90% propan-2-ol/10% acetonitrile. The gradient profile was: t=0.0, 32% B; t=1.0, 32% B; t=4.0, 45% B; t=5.0, 52% B, t=8.0, 58% b; t=11.0, 66% B; t=14.0, 70% B; t=18.0, 75% B; t=21.0, 97% B; t=25.0, 97% B; t=25.1, 32% B; t=30.0, 32% B. The flow rate was 0.26 mL/min, column temperature 45 °C, injection volume 10 µL. The run time of the method was 30 min.

In both assays parallel mass spectrometry experiments were performed collecting high resolution full scan (70,000 resolution) and top10 data dependent MS/MS fragmentation data (17,500 resolution) in both positive and negative ion mode in all injections (isQCs, standards and samples) at 3 normalised collision energies. The PMP mass range covered 70-1050 *m/z* with source voltage 3.5 kV, capillary temp. 380 °C, aux gas heater temp. 400 °C; gas flows: sheath gas 60 arbitrary units (au), aux gas 20 au, sweep gas 0 au, S-Lens RF Level: 50. The lipid mass range covered 120-1800 *m/z* with source voltage 3.5 kV, capillary temp. 250 °C, aux gas heater temp. 350 °C, gas flows: sheath gas 25 arbitrary units (au), aux gas 15 au, sweep gas 0 au.

Male and female samples were run in separate, randomised batches along with MTox700+ (Sostare et al. 2022) mixed reference standards adjusted for rat plasma for peak identification. System suitability testing was performed prior to each run start with acceptance criteria on retention time reproducibility, peak shape and mass accuracy of selected analytes of the mixed standard. A single merged data table was created for each sex by processing the four runs for both an untargeted (annotated MSI level 2-4) and a targeted readout (MSI level 1, MTox700+ toxicologically relevant metabolite panel).

The LC to MS transfer line blocked up and leaked during the female lipid run, therefore the 3rd aliquot of the provided plasma sample was used to repeat this batch. Two samples were excluded due to missed injection (1.1% loss in data).

Peak picking, peak alignment, background subtraction, gap fill and peak annotation was carried out using Compound Discoverer for PMP (Thermo v3.3) and LipidSearch for Lipidomics (Thermo, 4.2.29). Merged data table was generated using in-house workflows in KNIME (v4.5.1.). Test substance related features were removed by filtering the binned feature table (with annotations removed) for features that had larger than 20-fold change to control (dose responsive). In total 4016 and 4038 metabolites (binned features) were detected before the QC RSD<35% filtering was applied, resulting in 4% and 4.7% loss of the detected features (males and females respectively). 60% high-confidence annotation coverage was achieved (MSI level 1 or 2) with mass accuracy under 4 ppm and retention time drift under 0.2 min across the whole study. Response drift was assessed using PCA and drift correction was applied (R 4.1.1, Limma 3.48.3) on the log transformed data prior to data interpretation.

References

Kende A, Lai F, Lim P, et al (2023) Mode of action hypothesis testing in chemical safety assessments using metabolomics as supporting evidence: Phenobarbital and cyclobutrifluram metabolomics profile comparison. Toxicol Lett 382:13–21. https://doi.org/10.1016/j.toxlet.2023.04.008

Sostare E, Lawson TN, Saunders LR, et al (2022) Knowledge-Driven Approaches to Create the MTox700+ Metabolite Panel for Predicting Toxicity. Toxicological Sciences 186:208–220. https://doi.org/10.1093/toxsci/kfac007

Phenome Centre Birmingham, University of Birmingham (UoB)

Sample extraction. Plasma samples were thawed on wet-ice, briefly vortexed (5 s, full power, room temp.) and two aliquots (35 µL) were taken from each biological sample into two clean 2 mL microfuge tubes (Eppendorf): one to be prepared for polar metabolomics analysis, the other to be prepared for lipidomics analysis. To create the isQC samples, a further 35 µL was taken from each biological sample and pooled in a 7 mL glass vial with a Teflon lined cap (Wheaton). This pool was vortexed (30 s, full power, room temp) and aliquoted into multiple 35 µL aliquots in clean 2 mL microfuge tubes (Eppendorf). To prepare the process blank samples, 35 µL LC-MS grade water (VWR) was aliquoted into clean 2 mL microfuge tubes. Biological sample, isQC and process blank aliquots were stored at -80°C until extraction. Metabolites and lipids were extracted from the plasma samples using two monophasic protein-crash methods (Southam et al. 2020). First, plasma aliquots (35 µL) were thawed on ice. For polar metabolite extraction, 105 µL ice-cold polar organic solvent (acetonitrile/methanol, 50:50, v/v; LC-MS grade, VWR) was added to plasma (1/3 plasma/polar organic solvent, v/v) and each sample was vortexed (full power, 15 s, room temperature). Samples were centrifuged (20,000-g, 4 °C, 20 min) and 80 µL of the supernatant was loaded into a low recovery HPLC vial (Chromatography Direct, UK) and placed on the LC autosampler at 4 °C ready for analysis. For non-polar metabolite (lipid) extraction, 105 µL ice-cold non-polar organic solvent (100% isopropanol, LC-MS grade, Merck) was added to plasma (1/3 plasma/non-polar organic solvent, v/v) and each sample was vortexed (as above). Samples were centrifuged, and 80 µL of supernatant added to the LC autosampler, as for the polar samples. Preparation of the process blank and the isQC samples was carried out in the same way as the polar and non-polar metabolite extractions of the biological samples.

UHPLC-MS metabolomics. All samples were analysed applying two UHPLC−MS methods (HILIC and Lipid assays, see below) in positive and negative ion modes separately using a Dionex UltiMate 3000 UHPLC system coupled with a heated electrospray Q Exactive Focus mass spectrometer (Thermo Scientific). isQC samples were analysed as ten of the first eleven injections (to condition the analytical platform), and then every sixth injection was an isQC sample, with two isQC samples injected at the end of the analytical batch. A process blank sample was analysed as the sixth injection and the last injection of each batch. All data were collected as MS1 data in profile mode except for QC samples where MS/MS data was collected for metabolite identification purposes. Two MS/MS approaches were used: (i) using the "Discovery" DDA setting but with a prioritised inclusion list containing an in-house library of metabolic biomarkers (MTox700+; Sostare et al. 2022); accurate *m/z* and retention time window of +/- 20 s from the library), and an exclusion list containing the 25 most intense ions in the mobile phase which have no retention behaviour; (ii) using the "Discovery" DDA setting with no inclusion list, and an exclusion list containing the in-house library (accurate *m/z* and retention time window of +/- 20 s from the library) and the 60 most intense ions in the mobile phase which have no retention behaviour. Both approaches (i) and (ii) were collected over 6 different *m/z* ranges in duplicate – once in the middle of the sequence and once at the end of the sequence. The different *m/z* ranges used were: HILIC: 70−140 *m/z*; 115−185 *m/z*; 150−220 *m/z*; 200-310 *m/z*; 300-510 *m/z*; 500-1050 *m/z*; and lipids: 150−300 *m/z*; 290-365 *m/z*; 355-460 *m/z*; 450-860 *m/z*; 850-1100 *m/z;* 1000-2000 *m/z.* Stepped normalised collision energies of 20, 40, 130% were used across all methods. All samples were maintained at a temperature of 4 °C during the analytical batch.

HILIC assays employed an Accucore150-Amide-HILIC column (100×2.1mm, 2.6µm, Thermo Scientific). For positive ion analysis, mobile phase A was 10 mM ammonium formate dissolved in acetonitrile/water/formic acid (95:4.9:0.1 (v/v) and mobile phase B was 10 mM ammonium formate dissolved in acetonitrile/water/formic acid (50:49.9:0.1 (v/v)). For negative ion analysis, mobile phase A was 10 mM ammonium acetate dissolved in acetonitrile/water/acetic acid (95:4.9:0.1 (v/v) and mobile phase B was 10 mM ammonium acetate dissolved in acetonitrile/water/acetic acid (50:49.9:0.1 (v/v)). The gradient elution applied was t=0.0, 1% B; t=1.0, 1% B; t=3.0, 15% B; t=6.0, 50% B; t=9.0, 95% B; t=10.0, 95% B; t=10.5, 1% B; t=14.0, 1% B. All changes were linear (curve = 5) and the flow rate was 0.50 mL/min. Column temperature was 35 °C and injection volume was 2 µL. Data were acquired in positive and negative ionisation mode separately (70 – 1050 *m/z*) with a mass resolution 70,000 (FWHM, *m/z* 200). Ion source parameters: sheath gas = 55 arbitrary units, aux gas = 14 arbitrary units, sweep gas = 4 arbitrary units, spray voltage = 3.2 kV (positive ion) / -2.7 kV (negative ion), capillary temperature = 380 °C, aux gas heater temperature = 440 °C. Thermo ExactiveTune (2.8 SP1, build 2806) software controlled the instruments and data acquisition. All data were acquired in profile mode.

Lipid assays employed a reversed-phase Hypersil GOLD C18 column (100×2.1 mm, 1.9µm; Thermo Scientific). Mobile phase A was 10 mM ammonium formate dissolved in acetonitrile/water/formic acid (60:39.9:0.1 (v/v) and mobile phase B was 10 mM ammonium formate dissolved in isopropanol/acetonitrile/water/formic acid (85.5:9.5:4.9:0.1 (v/v)). The gradient elution applied was t=0.0, 20% B; t=0.5, 20% B, t=8.5, 100% B; t=9.5, 100% B; t=11.5, 20% B; t=14.0, 20% B. All changes were linear (curve = 5) and the flow rate was 0.40 mL/min. Column temperature was 55 °C and injection volume was 2 µL. Data were acquired in positive and negative ionisation mode separately (150–2000 *m/z*) with a mass resolution 70,000 (FWHM, *m/z* 200). Ion source parameters: sheath gas = 48 arbitrary units, aux gas = 15 arbitrary units, sweep gas = 0 arbitrary units, spray voltage = 3.2 kV

(positive ion) / -2.7 kV (negative ion), capillary temperature = 380 °C, aux gas heater temperature = 450 °C. Thermo ExactiveTune (2.8 SP1, build 2806) software controlled the instruments and data acquisition. All data were acquired in profile mode.

Data processing. Vendor format raw data files were converted to mzML file format using ProteoWizard software (Chambers et al. 2012). Full scan (MS1) data deconvolution was performed by XCMS v3.12.0 (Smith et al. 2006). Suspected xenobiotic features (i.e., those related to the test substances) were removed if they passed all of the following filters: present in <50% of control samples; present in >50% of high dose samples; intensity fold-change between control and high dose of >10-fold higher in treated samples; and a trend test (William's, p<0.05) between feature intensity and dose. The remaining features were blank filtered (features <20x intensity of the blank removed), filtered according to their presence in the QCs (features present in <70% of the QC samples were removed), corrected for signal/batch effects, missing value filtered such that all samples and features retained <50% missing values, and filtered for technical quality (features with RSD of the QCs >30% were removed). Remaining samples were normalised (PQN, median samples as reference (Dieterle et al. 2006)). Missing values were imputed (kNN, 5 neighbours) and samples were glog scaled (Durbin et al. 2002; Parsons et al. 2007) using QC samples as a reference. Univariate analysis was conducted using the normalised peak matrix. Multivariate analysis was conducted using the normalised, imputed and glog scaled matrix. All filtering and processing steps and statistical analysis were conducted using the R/Bioconductor package structToolbox (Lloyd et al. 2021).

QA/QC. Major sources of analytical drift were assessed and optionally corrected to ensure that high quality peak intensity matrices were used for downstream data processing and statistical analysis. First, *m/z* drift as a function of injection order was assessed by calculating the *m/z* error of known features (i.e. sodium salt clusters and ubiquitously abundant metabolites) in each assay across the study. It was found that *m/z* drift was present in the Lipid assays for male and female rats, and positive and negative polarity datasets. Mass drift was therefore corrected using a cubic spline fit of mass error versus injection order for the known features. Next, retention time drift was assessed by manually checking the chromatographic peaks of known features across all samples within each assay, and it was found that retention time drift was not required for any assay. Finally, signal drift was assessed based on PCA of the QC samples and total peak area plots as a function of injection order, and a correction was applied to all assays using quality control-robust spline correction (QC-RSC) (Kirwan et al. 2013).

References

Chambers MC, Maclean B, Burke R, et al (2012) A cross-platform toolkit for mass spectrometry and proteomics. Nat Biotechnol 30:918–920. https://doi.org/10.1038/nbt.2377

Dieterle F, Ross A, Schlotterbeck G, Senn H (2006) Probabilistic quotient normalization as robust method to account for dilution of complex biological mixtures. Application in1H NMR metabonomics. Anal Chem 78:4281–4290. https://doi.org/10.1021/ac051632c

Durbin BP, Hardin JS, Hawkins DM, Rocke DM (2002) A variance-stabilizing transformation for gene-expression microarray data. Bioinformatics 18:S105–S110. https://doi.org/10.1093/bioinformatics/18.suppl_1.S105

Kirwan JA, Broadhurst DI, Davidson RL, Viant MR (2013) Characterising and correcting batch variation in an automated direct infusion mass spectrometry (DIMS) metabolomics workflow. Anal Bioanal Chem 405:5147–5157. https://doi.org/10.1007/s00216-013-6856-7

Lloyd GR, Jankevics A, Weber RJM (2021) struct: an R/Bioconductor-based framework for standardized metabolomics data analysis and beyond. Bioinformatics 36:5551–5552. https://doi.org/10.1093/bioinformatics/btaa1031

Parsons HM, Ludwig C, Günther UL, Viant MR (2007) Improved classification accuracy in 1- and 2-dimensional NMR metabolomics data using the variance stabilising generalised logarithm transformation. BMC Bioinformatics 8:234. https://doi.org/10.1186/1471-2105-8-234

Smith CA, Want EJ, O'Maille G, et al (2006) XCMS: Processing Mass Spectrometry Data for Metabolite Profiling Using Nonlinear Peak Alignment, Matching, and Identification. Anal Chem 78:779–787. https://doi.org/10.1021/ac051437y

Sostare E, Lawson TN, Saunders LR, et al (2022) Knowledge-Driven Approaches to Create the MTox700+ Metabolite Panel for Predicting Toxicity. Toxicological Sciences 186:208–220. https://doi.org/10.1093/toxsci/kfac007

Southam AD, Haglington LD, Najdekr L, et al (2020) Assessment of human plasma and urine sample preparation for reproducible and high-throughput UHPLC-MS clinical metabolic phenotyping. Analyst 145:6511–6523. https://doi.org/10.1039/D0AN01319F

Vrije Universiteit Amsterdam (VU)

Samples were randomised thawed on wet ice, briefly vortex, centrifuged and aliquoted (3 x 50 µL plasma). For polar metabolite extraction a 50 µl aliquot was extracted by adding 100 µL of ice-cold 50:50 methanol/acetonitrile, and 50 µL of internal standard mix. The sample was then vortexed for 15 s and centrifuged (1330-g, 4 °C, 10 min). For the lipid extraction a 50 µL aliquot was taken, 140 µL of ice-cold propan-2-ol (LC-MS grade) and 10ul internal standard (SPLASH® LIPIDOMIX®) were added. The sample was then vortexed for 15 s and centrifuged (13,30-g, 4 °C, 10 min). 50 µL aliquots of each study sample (n=180) were transferred to an HPLC vial for LC-MS. Samples were keep at -80 °C until analysis. All samples were prepared within two days. IsQC samples: 20 µL aliquot of each extracted sample were transferred to a larger tube to form an isQC pool simultaneously to sample aliquoting. All samples were kept on ice during extraction and aliquoting. The samples were prepared across two days, therefore the first isQC pool (1.8 mL) was kept at 4 °C, and the second isQC pool (1.8 mL) was added on day two. The final isQC pool (ca. 3.6 mL) was vortexed for 30 s, and multiple 50 µL aliquots (30) were transferred into HPLC vials and kept with the other samples at -80 °C until LC-MS analysis. IsQC samples were analysed after 6 samples, and blanks after 10 samples (including isQCs). Additionally, runs with pure methanol were performed after every 10 measurements (blanks and QCs included).

HILIC analysis. Ultrahigh performance liquid chromatography (UHLPC) was used for the analysis of polar metabolites using an Agilent 1290 infinity liquid chromatography system (Palo Alto, CA) coupled to a high-resolution quadrupole time-of-flight MS (QTOF, Compact, Bruker Daltonik, Bremen, Germany) with an electrospray ionization (ESI) source in positive and negative modes. A UHPLC filter holder Accucore-150-Amide-HILIC (Thermo Fisher Scientific, Germany) was connected to a Thermo Amide HILIC column (2.6 µm, ID 2.1 mm x 100 mm). The mobile phase conditions for positive mode consisted of: Solution A - 10 mM ammonium formate and 0.1% formic acid in 95% acetonitrile/water; solution B - 10 mM ammonium formate and 0.1% formic acid in 50% acetonitrile/water. Mobile phase flow rate was 0.5 mL/min. Column temperature: 35 ^{o}C. Mobile phase gradient profile: t=0.0, 1% B; t=2.1, 1% B; t=4.1, 15% B; t=7.1, 50% B; t=10.1, 95% B; t=11.0, 95% B; t=11.5, 1% B; t=15.0, 1% B; t=20, 1%. Amount of sample injected 2 µL. The mobile phase conditions for negative mode were: solution

A - 10 mM ammonium acetate and 0.1% acetic acid in 95% acetonitrile/water; solution B - 10 mM ammonium acetate and 0.1% acetic acid in 50% acetonitrile/water. Mobile phase flow rate: 0.5 mL/min. Column temperature: 35 ^{0}C. Gradient profile: t=0.0, 1% B; t=2.1, 1% B; t=4.1, 15% B; t=7.1, 50% B; t=10.1, 95% B; t=11.0, 95% B; t=11.5, 1% B; t=15.0, 1% B; t=20, 1%. Amount of sample injected 2 µL. The MS data were collected in MS acquisition mode with 2 spectra/s, with a mass resolution >25000 FWHM. The MS parameter settings were as follows: capillary voltages of ±5000 V and ±4000 V in positive and negative modes, respectively; end set plates of ± 500 V; nebulizer gas (N2) pressure of 4 bar; drying gas flow rate of 6 L/min; and the drying gas temperature of 200 °C and 250 °C in positive and negative modes, respectively. The *m/z* range in MS detection was from 60 to 2000 in positive mode, and from 50 to 2000 in negative mode. The MS calibration solution (sodium formate) was analysed before every injection.

Lipid analysis. Ultrahigh performance liquid chromatography (UHLPC) for analysis of lipids was performed using a Agilent LC coupled to a Kinetex® (2.6µm EVO C18 100 Å particle size of 2.6 µm, 2.1 mm x 100 mm). The mobile phase conditions for positive mode consisted of: A - 10 mM ammonium formate and 0.1% formic acid in 60% acetonitrile/water; B - 10 mM ammonium formate and 0.1% formic acid in 85.5% propan-2-ol / 9.5% acetonitrile / 5% water. Mobile phase flow rate was 0.4 mL/min. Column temperature: 55 ^{0}C. Mobile phase gradient profile: t=0.0, 20% B; t=1.6, 20% B, t=9.4, 100% B; t=15, 100% B; t=17, 20% B; t=25, 20% B. Amount of sample injected 2 µL. The mobile phase conditions for negative mode were: A - 10 mM ammonium formate and 0.1% formic acid in 60% acetonitrile/water; B - 10 mM ammonium formate and 0.1% formic acid in 85.5% propan-2-ol / 9.5% acetonitrile / 5% water. Mobile phase flow rate: t=0.0, 20% B; t=1.6, 20% B, t=9.4, 100% B; t=15, 100% B; t=17, 20% B; t=25.0, 20% B. Amount of sample injected 2 µL. The MS data were collected in MS acquisition mode with 2 spectra/s, with a mass resolution >25000 FWHM. The MS parameter settings were as follows: capillary voltages of ±5000 V and ±4000 V in positive and negative modes, respectively; end set plates of ± 500 V; nebulizer gas (N_2) pressure of 4 bar; drying gas flow rate of 6 L/min; and the drying gas temperature of 200 °C and 250 °C in positive and negative modes, respectively. The *m/z* range in MS detection was from 60 to 2000 in positive mode, and from 50 to 2000 in negative mode. The MS calibration solution (sodium formate) was analysed before every injection.

Male and female were analysed in one sequence for polar or lipid analysis in positive or negative mode and samples were randomised before analysis.

Data processing. Internal mass calibration of each file was performed with injected sodium formate adducts. Data files were imported in MetaboScape (Bruker) for peak alignment and peak picking. Male and female data were processed separately. The negative mode data showed multiple samples with large Rt shifts in (both HILC and C18 lipids), and therefore the negative mode data was not used for grouping. Data were transferred to Excel for data filtering. Filtering of features was based on: 1) Features of the isQC with an RSD>30% were removed, 2) procedural blank (all features <5x blank were removed), 3) test substance specific features were removed, 4) features with less than 60% of data in any of the TS groups were removed, and 5) tolerable percent of missing values was set to 20%, max number of missing data allowed in a sample was 80%. As imputation method for missing estimations KNN (K=5) was used (Noreva 2.0; Fu et al. 2022). Feature drift correction was based on a Loess regression model using the isQC data using Noreva 2.0. Data was Log transformed, and normalisation of the samples was done with EigenMS (Noreva2.0). After pre-processing the following number of

features were selected; HILIC+: 1118 and Lipid RPC+: 428 for females, and HILIC+: 986 and Lipid RPC+: 92 for males.

References

Fu J, Zhang Y, Wang Y, et al (2022) Optimization of metabolomic data processing using NOREVA. Nat Protoc 17:129–151. https://doi.org/10.1038/s41596-021-00636-9

RESULTS AND DISCUSSION

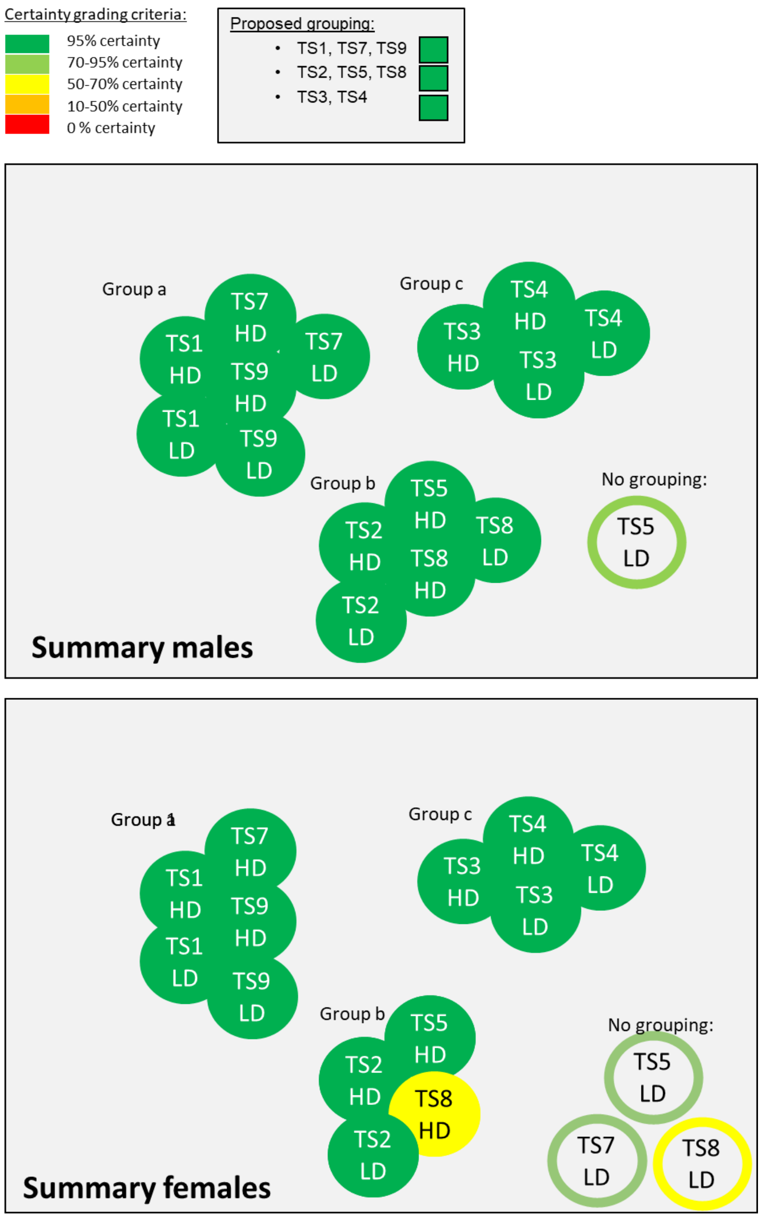

Figure S2. Results from the blinded analysis of the metabolomics data using the MetaMap®Tox database (following the BASF standard protocol for toxico-metabolomics, both LC-MS and GC-MS data were acquired by BMS and analysed by BASF SE), revealing that the eight test substances lie within three distinct groups. The colour scheme denotes the level of certainty in the grouping: if no grouping was possible due to too low effects on the metabolome, the substances are shown in unfilled circles. Key: LD – low dose, HD – high dose. These findings were provided to an unblinded scientist in the BASF SE team who could confirm that the rat plasma samples yielded the anticipated grouping of test substances as defined in Table 1.

Section S3. Intrastudy QC results

This section presents the conclusions reached by each partner on the quality of their processed metabolomics data. Organisation names have been replaced with **ring-trial partner (RP) numbers** to anonymise the identities of the partners' results. Individual partner statements are presented first, and Table S5 compares the key metric of median relative standard deviation (RSD) of feature intensities measured in the intrastudy quality-control (isQC) samples.

RP1 - After data processing, data quality was assessed using a combination of uni- and multivariate statistics for the detection of abnormalities. The study passed our QA/QC criteria for chemical grouping for both female and male rats.

RP4 - After data processing (including filtering and correction steps), the PCA scores plots and median RSD values were reviewed, and all assays were deemed to be of sufficient quality to continue to chemical grouping.

RP5 - In initial QA/QC, RSD values of internal standards in isQC samples across all three assays were less than 10%. After pre-processing (including run order correction and feature filtering), data quality was further assessed by PCA and RSD in isQC samples. Median RSD values for isQC samples were as follows: HILIC+: 8%; Lipid RPC+: 3%; Lipid RPC-: 4%. All assays were of sufficient analytical data quality to proceed to chemical grouping.

RP6 - After filtering and drift correction, the median isQC RSD was 9% and 15%, for male and female rats, respectively. The study passed our QA/QC criteria for chemical grouping.

RP7 - After processing, the median isQC RSD was 12% and 15% (males and females, respectively) for HILIC LC-MS, and 12% and 16% for the lipids analysis (males and females, respectively). The study passed our QA/QC criteria for chemical grouping.

RP8 - After data processing, including all filtering and correction steps, median RSD values of all feature intensities across all isQC samples were 24% (lipids) and 40% (polar metabolites). Additionally, PCA scores plots from global analyses of all the biological and isQC samples (for lipids and polar metabolites separately) failed to show tight clustering of the isQC samples for either dataset. Given the high levels of analytical variability displayed in these quantitative and qualitative assessments, the metabolomics data did not pass our QA/QC criteria.

Table S5. Quality assessment of metabolomics data by each ring-trial partner (RP) showing the median relative standard deviation (RSD) of feature intensities measured in the intrastudy quality-control samples (or for one partner, intralab QC samples) and in the biological study samples (where the biological samples correspond to those shown in the exemplar PCA scores plots in Figure 2, main paper). The much higher technical variability observed by partner RP8, compared to the other partners, is clearly evident. The data included for the biological samples is partner-protocol specific: (RP1) all LC-MS assays, male animals only; (RP4) one HILIC LC-MS assay, female animals only; (RP5) one C18 LC-MS assay, both sexes of animals; (RP6) all LC-MS assays, male animals only; (RP7) one HILIC LC-MS assay, female animals only; (RP8) two C18 LC-MS assays, both sexes of animals.

**Median RSD values (%)**	**RP1**	**RP4**	**RP5**	**RP6**	**RP7**	**RP8**
Intrastudy QC	8%	13%	4%	9%	12%	24%
Biological samples	39%	35%	53%	50%	21%	45%

The results in Table S5 can be interpreted as follows:

- As expected, the technical variability (described by median RSD of isQC) is considerably lower than the total technical and biological variability (described by median RSD of biological samples) for all six ring-trial partners.
- While the biological variability differs between partners, no partner has reported an excessively high value, and the values are in line with our expectations for mammalian plasma. Note that two of the largest values of 53% and 45% result from RP5 and RP8 who include both male and female rats in their calculations, whereas RP1, RP4, RP6 and RP7 present variation in plasma of a single sex.
- In terms of assessing the technical quality of each dataset, the median RSD of isQC is the critical measurement, and it clearly indicates high variability in the RP8 dataset. While there are different protocols for generating median RSD values for the isQC, RP8's value of 24% is approximately twice (or more) as high as all of the other ring-trial partner values.

Section S4. Analysis of metabolomics data to group chemicals

This section describes the univariate and multivariate statistical methods used by the partners for grouping the substances and estimating uncertainty. Organisation names have been replaced with **ring-trial partner (RP) numbers** to anonymise the identities of the partners' results.

RP1

As (a) the metabolome of female and male rats is known to differ and (b) the impact of a treatment can vary significantly between sexes, all analyses were done for each sex separately. All modelling and evaluation was done in R with packages specified. The first critical question for the classification task was the number of classes to be expected in the dataset. For this, the R package "factoextra" was used in combination with the imputation of missing values based on the nipals algorithm implemented in the "mixOmics" R package. To determine the optimal cluster number the "elbow method" was used with (a) kmeans and (b) hierarchical clustering (hca(cut)) with maximum expected cluster number set to 10 (there is a total of 17 groups). For both kmeans and hca(cut) analysis, the recommendation was to assume 4 clusters for both females and males. This matched with the observations from the "Bootstrap PCA", an approach that generates pseudo-samples by within-group bootstrapping. To generate artificial samples, a bootstrap sampling algorithm was used. A pseudo-animal metabolic profile was generated by independently sampling, with replacement, individual metabolite data restricted within a treatment group, stratified by sex, and dose. The resulting metabolic profile contains, for each metabolite, a value that is randomly selected from one of the animals of the respective treatment group. This procedure was repeated 100 times for each group to get 100 pseudo-animals that were split into 70 training and 30 testing samples. The sample procedure reduces the impact of outliers. As each metabolite is independently sampled, it breaks any inter-metabolite correlations, but as the sampling is restricted within a group it preserved inter-group variabilities. A PCA run with these amplified pseudo-samples reduces the intragroup variability making the intergroup differences more obvious (Figure RP1.1).

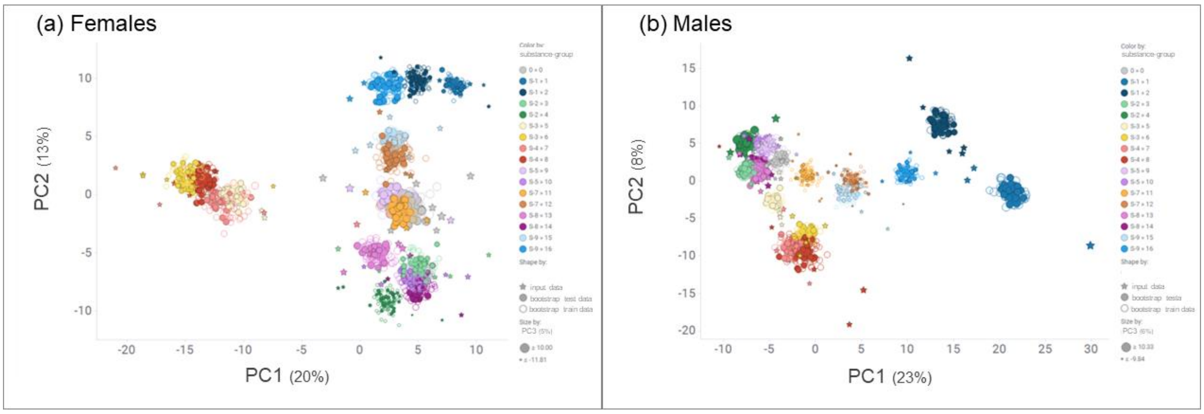

Figure RP1.1. Bootstrap PCA for females (a) and males (b). Per sex, for each of the groups, 100 pseudo-samples were generated by bootstrapping and split into 70 pseudo-samples for training and 30 for testing. Using the model generated based on the training set, the test set as well as the original input data were predicted.

For females, 3 clusters were separated from the controls while some treatments co-clustered with the controls in a fourth cluster. For the males, it was less clear whether 3 or 4 clusters would represent the data best. Also here, some treatments co-clustered with the controls. It was decided that the analysis should start with classification into 4 clusters used for all subsequent evaluations unless specified otherwise.

The full data evaluation was based on a combination of 5 different multivariate approaches used to derive co-clustering assignments between the 16 dose groups (2 dose groups for 8 test substances) each for males and females. Due to the limited information available for supervised analyses, only unsupervised methods were applied using different input levels (t-values, group medians, single sample profiles) to address different aspects with the aim to get a balanced analyses portfolio. The concept was to run several independent evaluations to be combined in the end to generate a "majority vote". The approaches used were: (a) Pairwise correlation of t-value profiles, (b) Hierarchical clustering of median based profiles, (c) Hierarchical clustering of sample profiles, (d) "Bootstrap PCA" (see above) using all dose groups and (e) "Bootstrap PCA" only using high dose plus control. In case of the "Bootstrap PCAs", the scores from the first three principal components were used for hierarchical clustering. All approaches were evaluated for the co-clustering of the different treatment groups resulting per sex in five 16x16 binary matrices where a score of 1 was representing co-clustering of two treatments and a score of 0 no co-clustering.

Most of the clusters obtained were calling low and high dose treatments of the same substance also in the same cluster (as expected). However, there were exceptions, especially for TS7 in females. In addition, the number of significant changes for TS5 were in the range of expectation by chance. At the time of the blinded evaluation, it was impossible to say which approach was most promising. Therefore, for the final evaluation, the five 16x16 co-clustering matrices were aggregated into one by summing up the scores (maximal possible result is 5, minimum is 0). This aggregated matrix was then used as input for hierarchical clustering for the final evaluation of the majority vote-based clustering result (Figure RP1.2). At this point also the number of clusters was re-evaluated with a focus on the high dose grouping. For females, when a split into 4 clusters was applied (not shown), the LD treatments (TS5 LD and TS7 LD) were forming a separate cluster, suggesting that both treatments were comparably weaker.

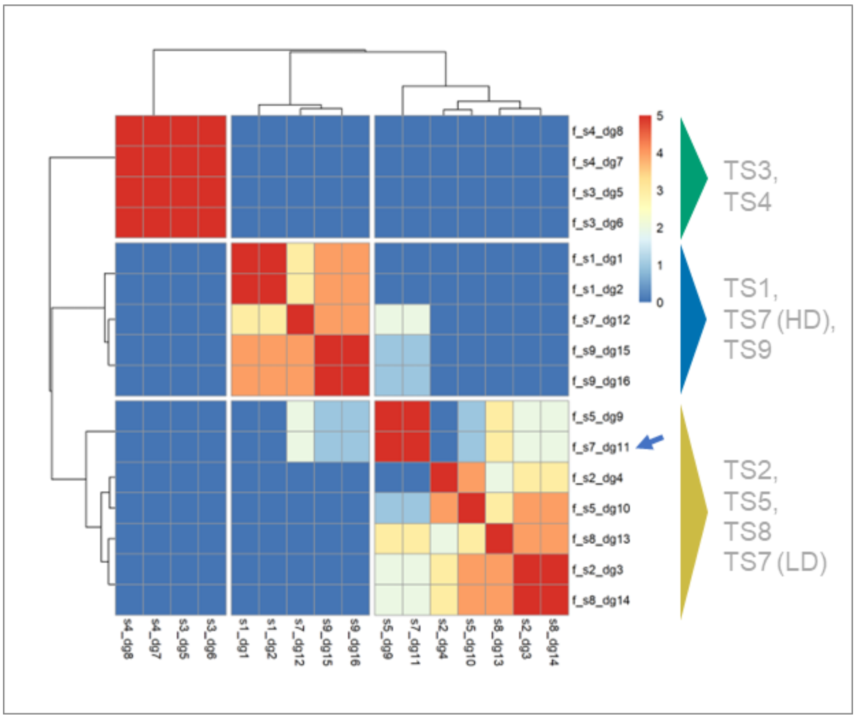

Figure RP1.2. Hierarchical clustering of the aggregated co-clustering results obtained based on the 5 different multivariate evaluations. Shown are the results for females. The representation of the test substances in the 3 clusters is indicated on the right.

For males, all high and low doses of the test substances were represented in the same cluster. In the 4 cluster scenario (not shown), TS1 (LD and HD) was forming a separate cluster. As it was assumed that the number of groups was identical for females and males, the grouping of the dataset into 3 clusters was considered most appropriate (Group A: TS1, TS7, TS9; Group B: TS2, TS5, TS8; Group C: TS3, TS4). In this 3 cluster scenario, for males, TS1 co-clustered with TS7 and TS9 with lower within cluster similarity compared to the other clusters suggesting that the TS1 treatment was stronger (as also supported by the Bootstrap PCA, see RP1.1b).

RP4

t-statistics were computed for all features comparing each substance and dose combination with the control samples. The proportion of features (across all four assays, males and females separately) showing a significant difference ($q < 0.1$) was used as a proxy for the magnitude of response. Any dose group of a test substance with a low percentage (threshold set to <1%) of features showing a significant difference vs the controls was excluded as having a weak overall response that would be difficult to group reliably, and potentially could inhibit the grouping of other substances (Table RP4.1). On this basis, TS5 low dose (0.01% of features changed significantly) and TS7 low dose samples (0.87%) were excluded from grouping of the female samples.

Table RP4.1. Percentage of significantly changing metabolic and lipid features (summing across all 4 UHPLC-MS assays) following exposure to test substances (TS) at low or high dose.

Test substance code	Female rats		Male rats	
	Low dose	High dose	Low dose	High dose
TS1	28.16%	29.16%	38.06%	42.06%
TS2	8.68%	16.05%	22.87%	31.83%
TS3	34.46%	41.01%	25.88%	31.85%
TS4	16.24%	34.85%	30.10%	18.92%
TS5	0.01%	6.65%	4.56%	19.68%
TS7	0.87%	10.15%	18.88%	27.99%
TS8	8.61%	9.18%	19.90%	19.57%
TS9	12.13%	22.56%	26.11%	38.92%

Consensus PCA was applied to all assays simultaneously (all remaining doses, male and females separately) after applying block-scaling to account for different amounts of variability in each assay (Figure RP4.1). Clear groupings (Group A: TS1, TS7, TS9, and Group C: TS3, TS4) were observed for both males and females. A third grouping (Group B: TS2, TS5, TS8) was more difficult to identify due to weaker response for TS5 and TS8 even in the high dose samples, and this was particularly evident for the female samples. However, both the male and females showed a similar overall grouping of substances, though the grouping was more distinct in the male samples. The three hypothesised groups, which were the same for both males and females, were also evident using HCA applied to t-statistics comparing each dose to the control group for all features (Figure 3B). Bootstrapping was used to indicate confidence in the HCA and resulted in confidence values >95% for all groups.

To further enhance confidence in the hypothesised groupings, two-group multi-block PLSDA models of each group vs the controls were trained using all assays and validated using 5-fold cross-validation and permutation testing (Figure RP4.2). All PLSDA models were able to discriminate between hypothesised groups and the control samples with low error rates in comparison to those observed for permuted models. Females and males were analysed separately; male results not shown.

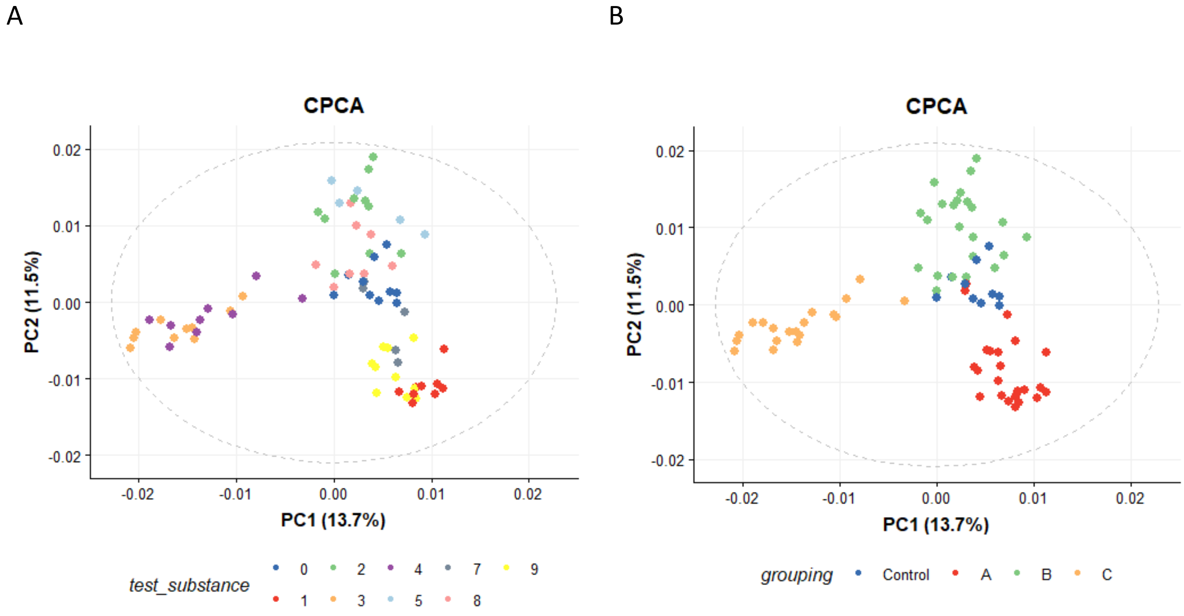

Figure RP4.1. Females CPCA scores plots for all assays combined. Low response groups and outliers were excluded. A) coloured by test substance and B) coloured by grouping result.

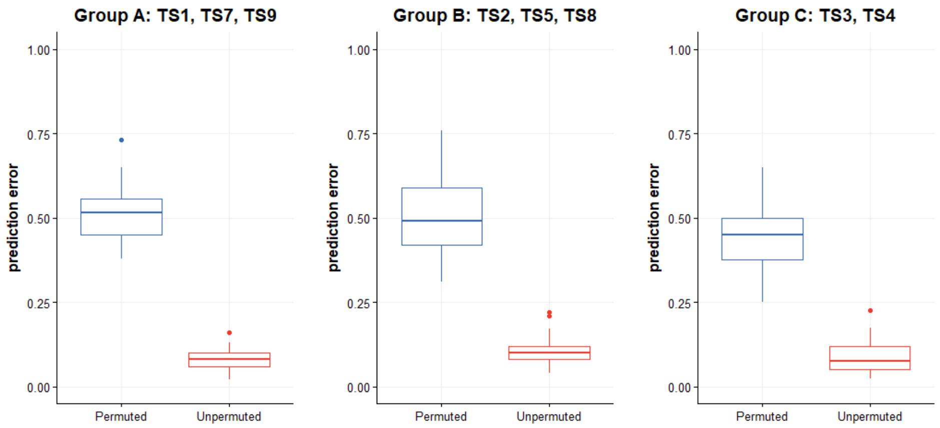

Figure RP4.2. 5-fold cross-validation prediction errors for multi-block PLSDA models for each of the hypothesised groupings, for the female samples, using unpermuted group labels (red). These models are compared to the prediction errors when using permuted group labels (blue), which represent a null model (i.e. one in which there is no grouping present). Results for males were similar (not shown).

RP5

To assess sample test substance (TS) grouping and grouping confidence, several approaches were applied, initially on the high dose group (DG2) samples. Data from female and male animals were modelled separately. All modelling was done in Python with libraries specified. Hierarchical cluster analysis (HCA) was performed using the scipy.cluster.hierarchy library (ward linkage; Euclidean distance) on average fold change to control data (average intensities calculated per DG/TS/sex sample subset, fold change calculated between average intensities of each TS1-9 and TS0) for each separate

assay (HILIC+, Lipid RPC-, Lipid RPC+; profiling and extracted target metabolites; 6 datasets in total). Results showed three clear clusters in both females and males, and across all assays (Figure RP5.1). These groups comprised: A, TS1, TS7 and TS9; B: TS2, TS5 and TS8, and C: TS3 and TS4.

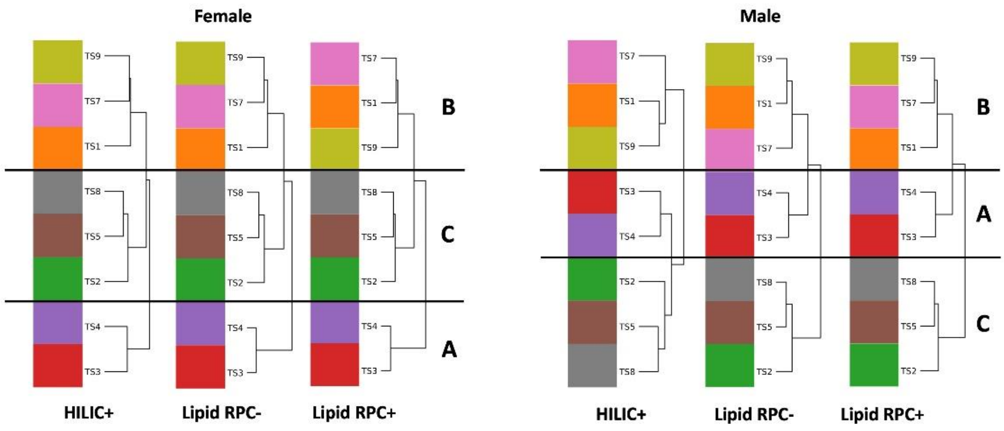

Figure RP5.1. Results of HCA for female and male animals (left and right panels respectively) on global profiling data from each of the three assays.

In addition, t-tests were used as the basis to estimate profile similarity. For each variable, t-tests were calculated using the scipy.stats.ttest_ind library (equal_var=False) between each possible pair of test substance group samples for each assay separately (HILIC+, Lipid RPC-, Lipid RPC+; profiling and extracted target metabolites; 6 datasets in total). For each pairwise combination of TS groups the percentage shared profile was computed as the percentage of variables with p-value > 0.05. Finally summary statistics, including the mean and standard deviation in percentage shared profile were calculated across all datasets. Again, the same three clear groups were observed in both females and males (Figure 3C), although TS7 also showed some overlap with Group C (containing TS2, TS5 and TS8) in the female animals.

Finally, linear discriminant analysis (LDA) was performed using the sklearn.discriminant_analysis library (SVD solver) on a combined intensity dataset (including data from all assays, profiling data only). Here, samples were removed for one test substance at a time, the classifier was built on all remaining samples, and the removed class was predicted using the resulting model. High prediction accuracy for these observed groupings (A, B and C) were observed in both females and males (Figure RP5.2, left panels).

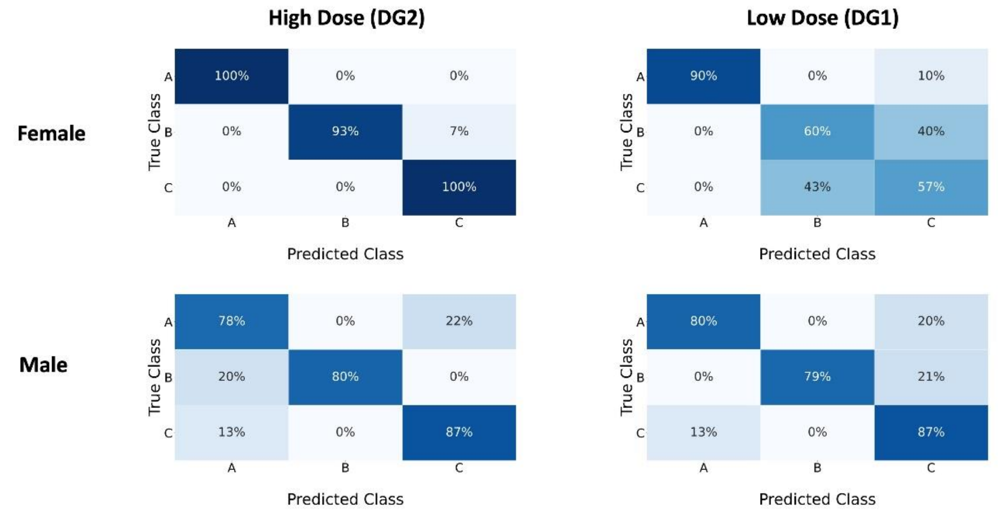

Figure RP5.2. Confusion matrices of results from LDA for female and male animals (top and bottom panels respectively) and high and low dose groups (left and right panels). True class is given in each row, with the percentage of these samples classified into each class shown.

When LDA models were repeated on low-dose samples (Figure RP5.2, right panels), similar results were observed, but with some ambiguity, expected owing to reduced metabolic effects for some test substances at the lower treatment dose. In a similar vein, when models (HCA and LDA) were repeated including control samples, the control group clustered with Group B (containing TS2, TS5 and TS8), again indicating a reduced metabolic impact of these test substances.

RP6

Principal Component Analysis was performed in SIMCA-P (Sartorius v17) on both the original (Figure RP6.1) and the bootstrapped data sets (Figure 3D) (R v4.1.1, Rsampling v0.1.1, mosaic v1.8.3, dplyr v1.0.8) which gave clear indication that three major effect trajectories were present in the data for both sexes.

To confirm the grouping observed in the PCA plots, we performed OPLS-DA comparing each test substance exposed group to the controls and used pairwise Shared and Unique Structure (SUS) plots to assess the similarity of the metabolomic profiles. To enhance our confidence in the grouping we used the OPLS-DA models for Differentially Abundant Metabolites (DAMs) selection (primary selection: p(corr)>|0.5| and VIP>1, followed by filtering and verification steps) and performed biochemical interpretation for the 8 test substances, producing a toxicological effect prediction for each of the observed groups and confirming the association of each test substance to the relevant group. The biochemical interpretation was especially helpful in reducing uncertainty in the correct grouping of TS5 (low responder) and TS7 (low/mixed responder), where observing consistent changes in key metabolites associated with the predicted toxicological mode of action supported the group assignment.

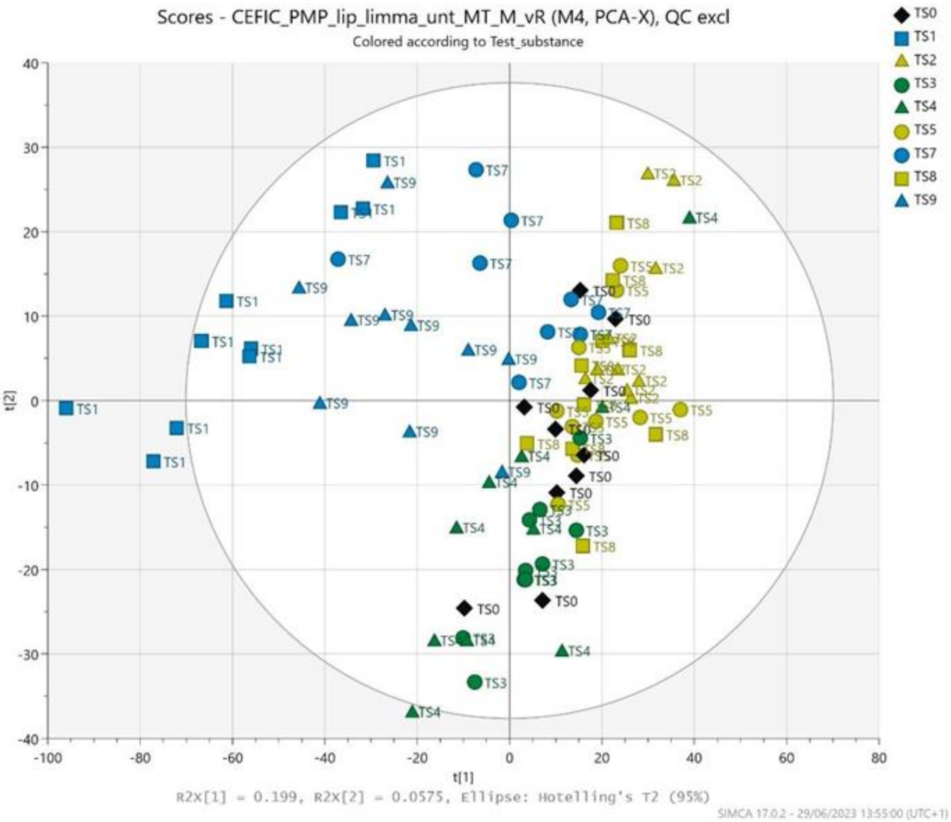

Figure RP6.1. Original PCA of male plasma samples (QCs removed).

RP7

Statistical analysis (MetaboAnalyst 5.0) was performed on the normalised data and HILIC and lipid female and male data were separately assessed. Principal Component Analysis (PCA) showed a clear grouping of the HILIC female and male samples in three groups. isQC samples showed low variation. Hierarchical cluster analysis (HCA) showed for all data three clear groups: A (Group A: TS3, TS4; group B: TS1, TS7, TS9; group C: TS2, TS5, TS8) (Figure RP7.1). Only the low dose of TS7 (TS7-DG1) showed overlap with group C, which included the control samples, but the high dose of TS7 grouped to B, and therefore TS7 was classified to group B. To further enhance the confidence in the grouping we performed Pearson correlations ($p<0.05$) between samples, and also here three groups were observed. Pearson correlations were also used as cut-off criteria for the grouping the HCA plots. The grouping was consistent between male and female. In addition, the HILIC and lipid data were combined for statistical analysis, and this further supported the grouping. TS3 and TS4 (Group A) had strong metabolite signatures, and the test substances in group C were low responders and were close to the control group.

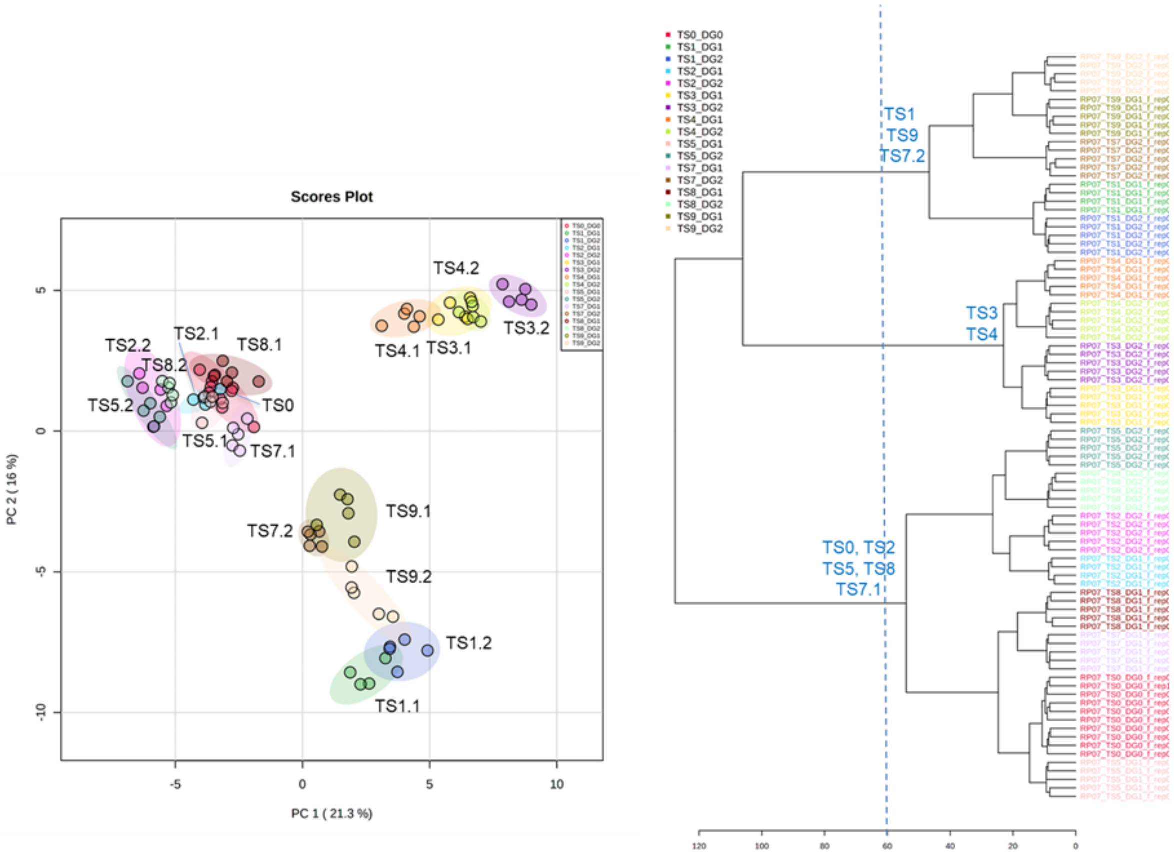

Figure RP7.1. Left: PCA analysis of the female HILIC data. Right: corresponding HCA plot, and the cut between the three groups was based on Pearson correlation analysis.

RP8

Metabolomics data did not pass QA/QC, no grouping results to describe.
