# Supplementary material for: Demonstrating the reliability of in vivo metabolomics based chemical grouping: towards best practice
Source: Arch Toxicol. 2024 Feb 18;98(4):1111–23. doi: 10.1007/s00204-024-03680-y (PMC10944399; doi:10.1007/s00204-024-03680-y)
Supplement: Supplementary file 2 — Supplementary file2 (PDF 98 KB) [file 204_2024_3680_MOESM2_ESM.pdf]

Table S6. Collation of methods used by the ring-trial partners for metabolomics data processing and bioactivity-based chemical grouping. Methods are structured according to the modules of the new OECD Omics Reporting Framework (Harrill et al., 2021), specifically the 'Data Acquisition and Processing Reporting Module for Mass Spectrometry' (DAPRM - MS), the 'Data Analysis Reporting Module for Multivariate Analysis (DARM - MVA), and the draft 'Chemical Grouping - Application Reporting Module' (CG - ARM).

High-level		Mid-level			Low-level		
**Description**	**Number of partners**	**OORF Reporting Module**	**OORF Reporting Element**	**Number of partners**	**Reported by at least one partner**	**Low-level**	**Number of partners**
Sample processing	6	DAPRM - MS	Extraction method general description	6	Polar - methanol (MeOH)/acetonitrile (ACN), Polar - ACN, Lipid - isopropanol (IPA), Combined - MeOH/dichloromethane/water/toluene	Polar - methanol (MeOH)/acetonitrile (ACN)	4
						Polar - ACN	1
						Lipid - isopropanol (IPA)	5
						Combined - MeOH/dichloromethane/water/toluene	1
		DAPRM - MS	Extract concentration and reconstitution in solvent for mass spectrometry analysis	6	Polar - samples did not require drying and reconstitution, Lipid - samples did not require drying and reconstitution, Lipid - sample concentration and solvent exchange	Polar - samples did not require drying and reconstitution	6
						Lipid - samples did not require drying and reconstitution	5
						Lipid - sample concentration and solvent exchange	1
Data acquisition	6	DAPRM - MS	QC samples	6	Intrastudy QC from pooled sample aliquots (male & female together), Intrastudy QC from pooled sample extracts (male & female together), Intrastudy QC from pooled sample extracts (male, female separately), Intralab pooled QC (male & female together)	Intrastudy QC from pooled sample aliquots (male & female together)	2
						Intrastudy QC from pooled sample extracts (male & female together)	2
						Intrastudy QC from pooled sample extracts (male, female separately)	1
						Intralab pooled QC (male & female together)	1
		DAPRM - MS	Process blank sample	6	Process blank filtering	Process blank filtering	6 (dictated by ring-trial protocols)
		DAPRM - MS	Mass spectrometry assay type	6	Targeted with relative quantification, Untargeted with relative quantification, Hybrid with relative quantification, Hybrid with relative quantification, targeting MTox700+ biomarkers	Targeted with relative quantification	1
						Untargeted with relative quantification	2
						Hybrid with relative quantification	1
						Hybrid with relative quantification, targeting MTox700+ biomarkers	2
		DAPRM - MS	Instrument configuration(s) and method(s)	6	Polar - HILIC LC-MS (pos & neg ion modes), Polar - HILIC LC-MS (pos ion mode), Lipid - C18 LC-MS (pos & neg ion modes), Lipid - C8 LC-MS (pos & neg ion modes)	Polar - HILIC LC-MS (pos & neg ion modes)	5
						Polar - HILIC LC-MS (pos ion mode)	1
						Lipid - C18 LC-MS (pos & neg ion modes)	5
						Lipid - C8 LC-MS (pos & neg ion modes)	1
		DAPRM - MS	Acquisition order for QC samples, biological samples and reference standard samples	6	Male & female animals separated & randomised, Male & female animals separated & block randomised, All samples randomised, All samples block randomised	Male & female animals separated & randomised	1
						Male & female animals separated & block randomised	1
						All samples randomised	2
						All samples block randomised	2
Data preparation	6	DAPRM - MS	Centroiding, baseline correction and noise reduction	6	MultiQuant, Centroiding	MultiQuant	1
						Centroiding	5
		DAPRM - MS	Data reduction	6	MultiQuant, manual inspection, Compound Discoverer, LipidSearch, MetaboScape, XCMS	MultiQuant	1
						manual inspection	1
						Compound Discoverer	1
						LipidSearch	1
						MetaboScape	1
						XCMS	3
		DAPRM - MS	Feature intensity drift and/or batch correction	6	Within-batch ultrapool normalisation, Intra-study QC fit, Linear modelling	Within-batch ultrapool normalisation	1
						Intra-study QC fit	4
						Linear modelling	1

Data cleaning	6	DAPRM - MS	Identification and removal ("filtering") of features	6	Missing value filtering, dilution series correlation, process blank filtering, QC-RSD filtering, xenobiotic filtering, binning metabolite features, void volume filtering, minimum fraction filtering, sample/QC RSD	Missing value filtering	4
						dilution series correlation	2
						process blank filtering	5
						QC-RSD filtering	5
						xenobiotic filtering	5
						binning metabolite features	1
						void volume filtering	1
						minimum fraction filtering	1
						sample/QC RSD	1
		DAPRM - MS	Identification and removal ("filtering") of outlying samples	6	Technical reasoning, PCA visual inspection, PCA DModX, PCA Hotelling T2, missing value filtering	Technical reasoning	1
						PCA visual inspection	3
						PCA DModX	1
						PCA Hotelling T2	4
						missing value filtering	1
Data preprocessing	6	DAPRM - MS	Normalisation	5	Within-batch ultrapool normalisation, EigenMS, probabilistic quotient normalisation, unit normalisation, factor-level averaging, fold change relative to control	Within-batch ultrapool normalisation	1
						EigenMS	1
						probabilistic quotient normalisation	1
						unit normalisation	1
						factor-level averaging	1
						fold change relative to control	1
		DAPRM - MS	Missing value imputation	5	k-nearest neighbours, gap filling, half minimum value, NIPALS	k-nearest neighbours	3
						gap filling	1
						half minimum value	1
						NIPALS	1
		DAPRM - MS	Normality testing, scaling and/or transformations	6	Log transform, unit-variance scaling, generalised log transform	Log transform	4
						unit-variance scaling	4
						generalised log transform	1
Data quality assessment	6	DAPRM - MS	Intrastudy QC precision report	6	Median RSD of intrastudy QCs, Median RSD of intralab QCs, PCA scores plot of QC and biological samples	Median RSD of intrastudy QCs	5
						Median RSD of intralab QCs	1
						PCA scores plot of QC and biological samples	6
Metabolite feature annotation	5	DAPRM - MS	Processing methods for metabolite annotation	5	Commercial standards, public databases, theoretical m/z, LipidSearch, peakPanther, MetaboScape, Compound Discoverer	Commercial standards	5
						public databases	1
						theoretical m/z	1
						LipidSearch	2
						peakPanther	1
						MetaboScape	1
						Compound Discoverer	2
Bioactivity-based grouping	5	DARM - MVA	Unsupervised	5	HCA, Correlation, PCA, Bootstrap PCA, Consensus PCA	HCA	5
						Correlation	1
						PCA	6
						Bootstrap PCA	2
						Consensus PCA	1
		DARM - MVA	Supervised	5	HCA, PLSDA, OPLSDA, LDA, SUS plots, Correlation, Bootstrapping	HCA	1
						PLSDA	1
						OPLSDA	2
						LDA	1
						SUS plots	1
						Correlation	1
						Bootstrapping	1
Report grouping results	5	CG-ARM	Grouping results	5	Bioactivity-based grouping, Plausible toxicological interpretation	Bioactivity-based grouping	5
						Plausible toxicological interpretation	1
